# Pretreatment CALLY index as promising novel biomarker in the prediction of surgical and oncological outcomes in esophageal cancer: a multi-center retrospective cohort study

**DOI:** 10.3389/fimmu.2025.1605067

**Published:** 2025-05-21

**Authors:** Peize Meng, Tongtong Gu, Jiayong Xu, Haihua Huang, Hansong Jin, Yuchen Wang, Hang Zhang, Zheng Ruan

**Affiliations:** ^1^ Department of Thoracic Surgery, Shanghai General Hospital, Shanghai Jiao Tong University of Medicine, Shanghai, China; ^2^ Department of Pharmacy, Shanghai Sixth People’s Hospital Affiliated to Shanghai Jiao Tong University School of Medicine, Shanghai, China

**Keywords:** C-reactive protein-albumin-lymphocyte index, esophageal cancer, surgery, neoadjuvant therapy, overall survival, disease-free survival

## Abstract

**Background:**

Esophageal cancer (EC) is a global health challenge with high mortality rates. The traditional TNM staging system is limited in its ability to provide accurate prognostic predictions. This study aimed to investigate the utility of the C-reactive protein-albumin-lymphocyte (CALLY) index in the evaluation of mid- to long-term outcomes in patients undergoing esophagectomy.

**Methods:**

We conducted a multi-center retrospective cohort study of 657 EC patients admitted between 2010 to 2024, with 553 patients from Shanghai General Hospital (training cohort) and 104 from Shanghai Sixth People’s Hospital (validation cohort). Associations between the CALLY and overall survival (OS)/disease-free survival (DFS) were evaluated using multivariable-adjusted Cox regression analyses.

**Results:**

Patients with CALLY index > 2.55 were associated with significantly improved OS (adjusted hazard ratio [HR]: 0.55, 95% confidence interval [CI]: 0.43-0.71) and DFS (HR: 0.51, 0.40-0.65), independent of clinical risk factors. Incorporating CALLY index into clinical prediction models significantly enhanced discriminative ability (area under the receiver operating characteristic curve [AUROC] of OS: 0.719-0.752; AUROC of DFS: 0.745-0.788, P < 0.01). In the validation cohort, the same associations were also observed (HR of OS: 0.57, 95% CI: 0.42-0.78; HR of DFS: 0.53, 95% CI: 0.40-0.71). In both cohorts, CALLY index > 2.55 were associated with significantly reduced risk of recurrence.

**Conclusions:**

The CALLY index emerges as a cost-effective prognostic tool integrating inflammation-nutrition-immunity parameters. Its preoperative integration with tumor, node, and metastasis staging and other well-known risk factors might optimize risk stratification and guide personalized therapeutic strategies for EC patients undergoing esophagectomy.

## Introduction

Esophageal cancer (EC) remains one of the most prevalent and deadly malignancies worldwide, with a particularly high burden in Asia. According to the 2022 Globocan cancer statistics, Asia accounts for a significant proportion of global cases, with China alone representing 43.8% of new diagnoses and 42.1% of related deaths (1, 2). Surgical resection, the primary treatment for patients without contraindications, is often associated with severe complications such as anastomotic leaks, strictures, dysphagia, reflux, and malnutrition, which significantly affect patients’ quality of life and long-term prognosis. Despite advances in surgical techniques and rigorous tumor staging, the five-year survival rate for post-surgical EC patients remains below 25% (3). Therefore, more effective predictive tools are urgently needed to improve diagnostic accuracy and optimize treatment strategies.

The American Joint Committee on Cancer (AJCC) tumor, node, and metastasis (TNM) staging system is widely used for risk stratification and treatment planning (4). Despite its widespread clinical utility, the TNM staging system exhibits notable limitations that underscore the urgent need for more sophisticated prognostic tools. Specifically, its predictive accuracy is significantly compromised in forecasting long-term survival outcomes for patients undergoing neoadjuvant chemoradiotherapy (5). Moreover, the TNM staging fails to account for the substantial survival heterogeneity observed among patients with identical TNM staging (6), highlighting its limited capacity to capture the complex biological and molecular underpinnings of tumor progression. These critical shortcomings emphasize the imperative for developing more robust and comprehensive prognostic models that can better inform clinical decision-making and patient management. Recently, there has been a growing study on the discovery of innovative biomarkers, aiming to enhance the reliability of EC prognostic assessments. For example, IL-8, TIE2, and HGF have demonstrated strong correlations with overall survival (OS) and progression-free survival (PFS) in EC (7). However, the detection of these biomarkers often requires specialized kits, which are costly and not widely accessible, thus limiting their clinical utility. On the other hand, Elevated neutrophil-to-lymphocyte ratio (NLR) and platelet-to-lymphocyte ratio (PLR) have been reported in studies to correlate with adverse clinical outcomes in EC (8–10). Nevertheless, single or dual parameter biomarkers may lack the multidimensional capacity to capture the complex biological interplay underlying cancer prognosis. For instance, while Sun et al. identified high PLR as an independent predictor of reduced OS in EC, a meta-analysis by Ishibashi et al. (n=2,655 across seven studies) demonstrated no significant PLR-OS association (9, 11). These discrepancies, likely stemming from the limitations of single-dimensional biomarkers, underscore the critical need for composite indices.

To address these limitations, this study investigates the C-reactive protein-albumin-lymphocyte (CALLY) index, a composite biomarker that integrates C-reactive protein (CRP), albumin, and lymphocyte count to reflect a patient’s inflammatory, immune, and nutritional status. By combining these three key indicators, the CALLY index provides a comprehensive overview of a patient’s systemic health, offering valuable insights into their overall condition. Previous studies have established the CALLY index as an independent prognostic biomarker for long-term survival in postoperative colorectal cancer patients (12). Additionally, research by Katsunobu Sakurai et al. highlights its clinical utility in predicting OS and major complications in gastric cancer patients following gastrectomy (13). In the context of EC, existing studies have explored the correlation between the CALLY index and postoperative outcomes. However, these investigations are predominantly based on small, single-center datasets, which limit their sample representativeness and model generalizability. While these findings provide valuable preliminary insights, their restricted applicability across diverse healthcare settings underscores the need for more robust and widely generalizable research. This study aims to enhance the accuracy and universality of predictive models by investigating the relationship between the CALLY index and OS and disease-free survival (DFS) in EC surgical patients. Leveraging a specialized multi-center database with large-sample data, we seek to develop a more representative and reliable preoperative risk prediction model.

## Method

### Study population

Consecutive patients who suffered from EC and had received esophageal surgery were recruited from Shanghai General Hospital (SHGH) and Shanghai Sixth People’s Hospital (SHSH). Patients were excluded if they met the following criteria: (i) acute inflammatory state (e.g., infections such as pneumonia, acute cholecystitis, appendicitis, or sepsis, etc.); (ii) hematologic disease; (iii) immune system disorders; (iv) other malignancies or with other anti-cancer treatments; (v) incomplete medical records or missing data of CALLY index. This study was approved by the Ethics Committee of Shanghai General Hospital (reference no.2024KS492) and Shanghai Sixth People’s Hospital (reference no.2024-KY-205 (K)), following the standards for medical research in humans recommended by the Declaration of Helsinki. Written informed consent was obtained from all participants or their legal representatives.

### Data collection and variable definition

Patient cohorts were acquired from specialized esophageal cohort databases, which consist of patient information available from electronic health record (EHR) in digital format. We collected a series of variables, including patient demographics, admission assessment, comorbidities, laboratory test, surgical information, and histopathology. Missing values were filled in by a second manual review of the medical records, and patient personal information was de-identified before delivering for analysis.

As previously explained, CALLY index is calculated as albumin (g/L) × lymphocyte count (10^^9^/L)/CRP (mg/L). Blood samples were collected at 6 a.m. the day after hospital admission. This includes patients who are admitted to receive surgical treatment, as well as those who have received 1–2 cycles of neoadjuvant therapy before being accepted for surgical treatment. The tumor stage in the current study used the 8th AJCC/UICC TNM classification (6).

### Procedure and intensive care

The choice of surgery depends on the cancer’s location, stage, and the patient’s clinical condition. Sweet, Ivor-Lewis, and McKeown are the most commonly chosen surgical technique (14–16). Detail of surgery is provided in the **Supplementary Material**.

The surgical approaches and standard intensive care unit (ICU) protocols are approximately similar in both hospitals. Briefly, after the surgery was completed, patients were transferred to the ICU, placed on ventilators or not. Blood pressure, heart rate, respiratory rate, oxygen saturation, and drainage are recorded every 1 to 2 hours. Arterial blood gases were checked every 6 to 12 hours, depending on the patient’s condition. Postoperative care including respiratory support, pain management, fluid and electrolyte balance, infection prevention, cardiovascular monitoring, and nutritional support.

### Study outcome

The primary outcomes are OS and DFS, reported as time-to-event outcome. DFS is defined as the period of time from the completion of surgery to death from all causes or tumor recurrence, whichever occurs first. Post-operative follow-up included outpatient follow-up and phone calls. Patients were censored on August 31, 2024, or at the time of death from all causes. In the analysis of DFS, patients were censored at the time of recurrence or death from recurrence after surgery. Those who lost to follow-up were included using the last data recorded in the databases.

### Statistical analysis

Continuous variables are presented as means (standard deviation [SD]) when normally distributed or as medians (IQR) for variables with skewed distribution. Categorical data are presented as percentages. Statistical differences between continuous or categorical variables were established using a Student’s t test, Mann Whitney U test, chi-square test, or Fisher’s exact probability method as appropriate.

As most of the variables were collected through manual review of EHR, missing data was unavoidable. Our datasets are highly complete, with missing data ranging from 0% to 2.6%. Multiple imputation method was used to impute missing values (five imputations). The final dataset used for analysis was derived from the extraction of data across the five imputed datasets.

To enhance the general readability of the manuscript, we established the association between CALLY index levels and OS and DFS using multivariable-adjusted COX regression analyses, including CALLY index as a continuous or categorical variable dichotomized at 2.55. This cutoff value has been chosen based on three different cut point selection methods (Maximizing the Youden Index, Decision Survival Tree, and Non-restricted Spline Regression). Then using the backward stepwise regression method, we constructed three COX proportional hazards models for predicting OS including CALLY index dichotomized at three different cutoffs. Based on the lowest Akaike information criterion and Bayesian information criterion, we selected an optimal cutoff for CALLY of 2.55 for subsequent analyses.

Cox proportional hazards models for the association between CALLY index and OS were derived in all patients with CALLY ≤ 2.55 (n=257). Models were progressively adjusted for potential confounders. First, the model was adjusted for age and sex. Then, the model was adjusted for body mass index (BMI), hypertension, diabetes mellitus (DM), cerebrovascular accident, neoadjuvant chemotherapy, and tumor characteristics (ie, tumor location, histological type, differentiation, and TNM stage). In an exploratory model, additional adjustment for surgery information (duration of surgery, anastomosis site, intraoperative-red blood cell transfusion, and intra-operative blood loss) in the cohort was performed. We identified these potential factors based on clinical experience, statistical considerations, and previous literature reports. The association between CALLY index and OS and DFS was described using adjusted hazard ratios (HRs) for every 1 unit, 1 SD increase in CALLY index, and for CALLY index classification (CALLY index ≤ 2.55 vs. CALLY index > 2.55). The association between CALLY index and recurrence was performed using competing risk analyses in the Fine and Gray models, by treating death from other causes as a competing risk (17). This means censoring was applied at the time of unrelated death (i.e. death not preceded by tumor factor), or at the end of follow-up. The influence of the recurrence was assessed by deriving subdistribution HRs (SHRs) from the Fine and Gray models. In line with the primary analysis, SHRs from Fine and Gray models (including the same sets of potential confounders) were derived for every 1 unit, 1 SD increase in CALLY index, and for CALLY index classification.

A subgroup analysis was performed to explore the differences in OS and DFS across various demographic and tumor characteristics. The patients were divided into subgroups based on age (≤ 70 years vs. > 70 years), gender (male vs. female), histological type (esophageal squamous cell carcinoma [ESCC] vs. non-ESCC), and neoadjuvant therapy (yes vs. no). Additionally, an interaction effect analysis was conducted to assess the interplay between these variables.

Anastomotic complications (AC) in EC are important factors that affecting patient prognosis. Next, we investigated whether the association between CALLY index and OS/DFS was mediated by the occurrence of AC, by adjusting the AC as a time-varying covariate. Anastomotic complications are defined as anastomosis fistula, marginal ulcer, and anastomotic stenosis that need dilation treatment or stent implantation.

Reverse causality was assessed by repeating the analyses after excluding patients with OS and DFS within the first 0.5, 1, and 2 years after inclusion. Consistency of the association between CALLY and OS and DFS over time was assessed by determining the effects of CALLY within subsequent time intervals.

In addition, we explored the additional predictive value of CALLY index by adding it to the clinical risk model. We constructed a Cox regression model for OS and DFS by including well established risk factors (ie, age, gender, BMI, hypertension, DM, neoadjuvant chemotherapy, and radiotherapy, TNM stage, and tumor characteristics). Multicollinearity of the variables was evaluated and excluded by calculating variance inflation factor (VIF).

Furthermore, in order to evaluate the prognostic value of the CALLY index, we conducted a comparative analysis of CALLY index with other established systemic inflammation and immune-nutritional biomarkers, including the NLR, PLR, and the immune-inflammatory-nutritional score (IINS). NLR and PLR were calculated as follows: NLR = neutrophil count (10^^9^/L)/lymphocyte count (10^^9^/L); PLR = platelet count (10^^9^/L)/lymphocyte count (10^^9^/L). The IINS was computed by aggregating the values of CRP, lymphocyte count, and albumin. CRP (mg/L) was classified into three cohorts: score 0 (≤ 2.20), score 1 (2.20 < CRP ≤ 3.76), and score 2 (> 3.76). Lymphocyte count (10^^9^/L) classifications were: score 0 (> 1.98), score 1 (1.00 < lymphocyte count ≤ 1.98), and score 2 (≤ 1.00). Albumin (g/L) classifications were: score 0 (> 44.20), score 1 (35.40 < albumin ≤ 44.20), and score 2 (≤ 35.40) (18). The IINS was subsequently calculated by summing the scores of CRP, lymphocyte count, and lbumin, resulting in a range from 1 to 6. These biomarkers were selected for comparison due to their established roles in predicting cancer prognosis and their widespread clinical use (18–22). We assessed the discriminatory performance of CALLY, NLR, PLR, and IINS in predicting OS and DFS by incorporating each biomarker into the clinical models. This analysis aimed to determine whether CALLY index offers superior prognostic value compared to NLR, PLR, and IINS in the context of esophageal cancer patients undergoing esophagectomy. The area under the receiver operating characteristic curve (AUROC) was performed to evaluate the discrimination of the models. DeLong test, a non-parametric method based on standard error and covariance, was applied to compare the difference in AUROC between two curves.

Statistical analyses were performed using R (version 4.0.3, The R Foundation), with packages of *mice* (version 3.13.0), survival (version 3.3.1), *cmprsk* (version 2.2-10), *jstable* (version 1.3.5), *rms* (version 6.1-0), and *rmda* (version 1.6). A two-sided P value < 0.05 was considered statistically significant. Further details of the statistical analysis are provided in the **Supplementary Material**.

## Results

### Characteristics of the study population

Between January 1, 2016, and January 12, 2024, 553 patients were screened for enrollment in the SHGH. A flowchart of the patient enrollment process is provided in **Supplementary Figure S1**. The mean (SD) of their age was 64.8 (7.9) years (range: 36–85 years), and 456 (82.4%) of the patients were male. According to the TNM classification, 98 (17.7%), 275 (49.7%), 134 (24.2%), and 46 (8.3%) patients were in stages I, II, III, and IV, respectively. In patients with CALLY index ≤ 2.55 (n = 257), the median CALLY index level was 0.78 (IQR 0.40-1.50); while in patients with CALLY index > 2.55 (n = 296), the median CALLY index level was 7.38 (IQR 4.50-13.37); distribution is shown in **Supplementary Figure S2**. **Table 1** displayed the clinical characteristics and laboratory markers of patients with CALLY index ≤ 2.55 versus CALLY index > 2.55. The in-hospital outcomes between the two groups are shown in **Supplementary Table S1**. There were no significant differences in mortality, post-operative red blood cell transfusion, acute kidney injury, and hepatic insufficiency between the two groups (all P > 0.05, **Supplementary Table S2**).

**Table 1:** Clinical characteristics of patients with CALLY ≤ 2.55 vs. CALLY > 2.55.

Variable	CALLY ≤ 2.55 (n=257)	CALLY > 2.55 (n=296)	P value
Male	224 (87.2%)	232 (78.4%)	0.009
Age	65.3 ± 8.1	64.5 ± 7.9	0.264
BMI	22.6 ± 3.4	23.1 ± 3.3	0.092
Hypertension	69 (26.8%)	89 (30.1%)	0.458
DM	24 (9.3%)	18 (6.1%)	0.197
EF	64.0 (62.0, 66.0)	63.0 (62.0, 66.0)	0.467
Cerebrovascular accident	35 (13.6%)	40 (13.5%)	1.0
Neoadjuvant chemotherapy	23 (8.9%)	33 (11.1%)	0.475
Radiotherapy	5 (1.9%)	2 (0.7%)	0.257
Laboratory test			
White blood cell count	6.0 (5.0, 7.4)	5.4 (4.5, 6.5)	<0.001
Neutrophil count	3.9 (3.0, 5.0)	3.2 (2.6, 4.2)	<0.001
Lymphocyte count	1.4 (1.1, 1.7)	1.6 (1.3, 2.0)	<0.001
Monocyte count	0.4 (0.3, 0.5)	0.4 (0.3, 0.4)	<0.001
Red blood cell	4.3 (3.8, 4.6)	4.3 (3.9, 4.6)	0.558
Hemoglobin	131.0 (120.0, 143.0)	136.0 (124.0, 145.0)	0.018
Platelet count	213.0 (162.5, 268.0)	192.0 (154.0, 236.0)	0.001
C-reactive protein	6.8 (4.2, 13.4)	0.9 (0.5, 1.5)	<0.001
Serum creatinine	65.1 (58.8, 74.6)	66.8 (57.9, 77.0)	0.758
Blood glucose	5.0 (4.5, 5.8)	4.9 (4.5, 5.5)	0.230
Albumin	40.1 (36.8, 43.1)	40.8 (38.3, 44.3)	0.006
Alanine aminotransferase	14.3 (10.1, 19.6)	15.5 (12.0, 20.3)	0.021
Total bilirubin	12.7 (9.2, 16.4)	11.9 (9.0, 16.2)	0.407
Tumor markers			
NSE	12.3 (10.4, 15.2)	12.2 (10.4, 14.5)	0.555
CEA	2.1 (1.3, 3.3)	2.2 (1.5, 3.2)	0.367
AFP	2.8 (1.9, 3.8)	2.8 (1.9, 4.1)	0.635
CA125	9.7 (6.8, 13.5)	8.2 (5.9, 11.8)	0.003
CA199	10.0 (6.6, 17.3)	10.5 (5.7, 17.2)	0.350
Tumor site			0.654
Thoracic	217 (84.4%)	255 (86.1%)	
Abdomen	40 (15.6%)	41 (13.9%)	
Tumor type			0.979
Squamous cell carcinoma	212 (82.5%)	245 (82.8%)	
Adenocarcinoma	36 (14.0%)	40 (13.5%)	
Other types	9 (3.5%)	11 (3.7%)	
Degree of differentiation			0.532
0^a^	8 (3.1%)	6 (2.0%)	
Well	69 (26.8%)	94 (31.8%)	
Moderate	154 (59.9%)	165 (55.7%)	
Poor	26 (10.1%)	31 (10.5%)	
TNM stage			
T			0.011
T1	32 (12.5%)	69 (23.3%)	
T2	67 (26.1%)	72 (24.3%)	
T3	142 (55.3%)	141 (47.6%)	
T4	16 (6.2%)	14 (4.7%)	
N			0.610
N0	148 (57.6%)	183 (61.8%)	
N1	66 (25.7%)	74 (25.0%)	
N2	30 (11.7%)	29 (9.8%)	
N3	13 (5.1%)	10 (3.4%)	
M			0.201
M0	253 (98.4%)	294 (99.3%)	
M1	4 (1.6%)	2 (0.6%)	
Ki 67	50.0 (30.0, 70.0)	50.0 (30.0, 65.0)	0.233

Data are presented as means (SD), medians (IQR), or percentages.

a 1.Patients with no residual tumor after neoadjuvant therapy; 2.Patients who underwent endoscopic submucosal dissection (ESD) for esophageal lesions (pathologically confirmed as malignant), followed by radical esophagectomy, with no residual tumor found in postoperative pathology.

CALLY, C-reactive protein-albumin-lymphocyte index; CALLY, C-reactive protein-albumin-lymphocyte index; BMI, Body mass index; DM, Diabetes Mellitus; EF, Ejection Fraction; T, Tumor; N, Node; M, Metastasis; NSE, Neuron-Specific Enolase; CEA, Carcinoembryonic Antigen; AFP, Alpha-Fetoprotein; CA125, Carbohydrate Antigen 125; CA199, Carbohydrate Antigen 199; TNM stage, Tumor, Node, Metastasis stage.

**Figure 1:**
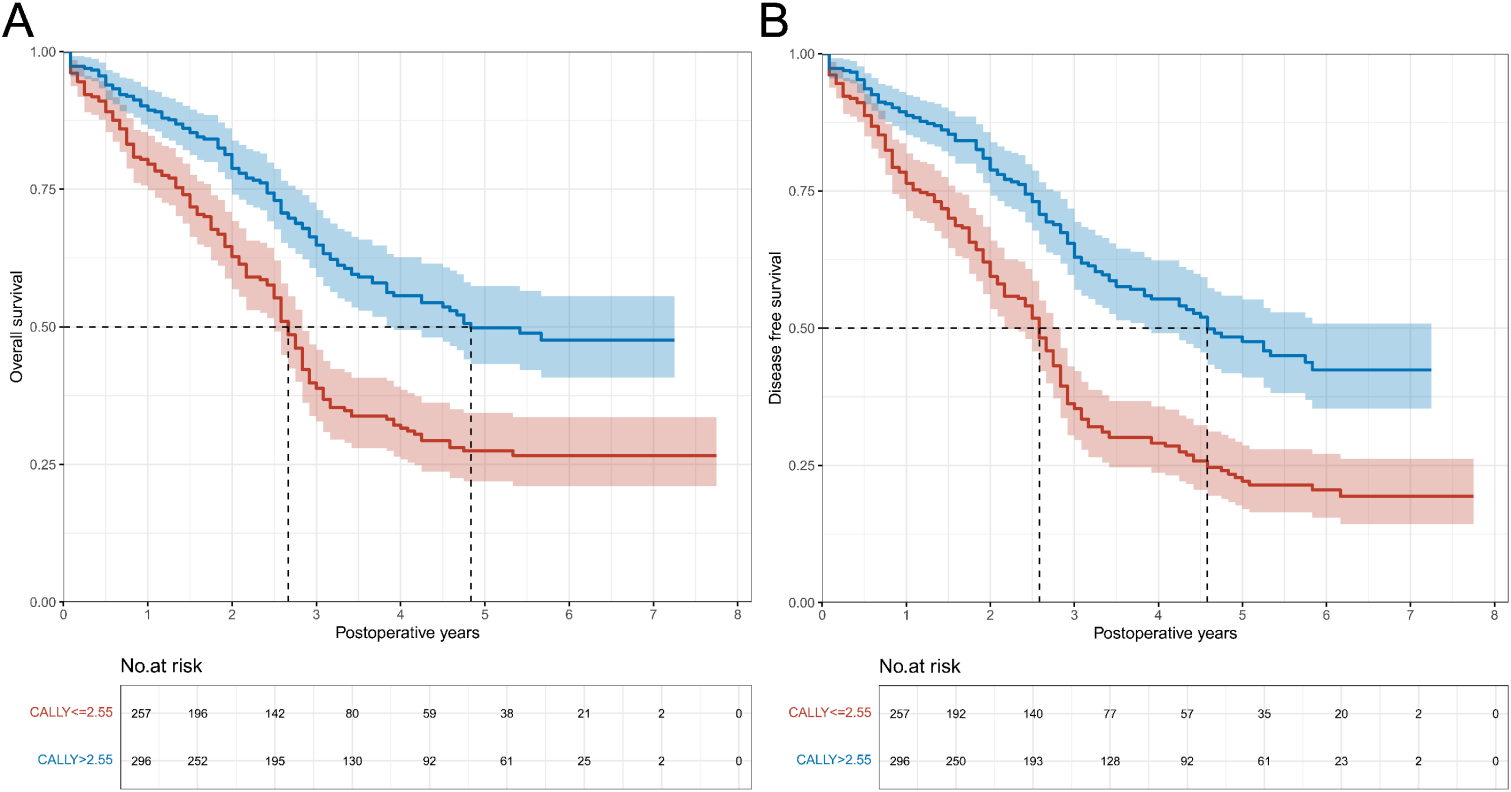
Overall survival and disease-free survival curves in patients with CALLY index ≤ 2.55 vs. CALLY index > 2.55 in the training cohort. **(A)** Comparison of overall survival curves between patients with CALLY index ≤ 2.55 and CALLY index > 2.55. **(B)** Comparison of disease-free survival curves between patients with CALLY index ≤ 2.55 and CALLY index > 2.55. CALLY, C-reactive protein-albumin-lymphocyte

Follow-up was completed in 95.3% of patients. Twenty-six individuals were lost to follow-up (4.7%), and no significant difference was found between the CALLY index groups (3.9% vs. 5.4%, P = 0.401). The follow-up time ranged from 1 to 93 months, with a median follow-up of 2.5 years (IQR: 1.3-4.3 years). 162 death cases were observed in patients with CALLY index ≤ 2.55 (63.0%; incidence rate: 24.74/100 person-years [PY]), and 115 cases in patients with CALLY index > 2.55 (38.9%; incidence rate: 12.85/100 PY). The median OS and DFS for patients with CALLY index ≤ 2.55 were 2.2 years (IQR: 1.0-3.7 years) and 2.2 years (IQR: 0.9-3.4 years), respectively; for patients with CALLY index > 2.55 mg/L, the median OS and DFS were 2.8 years (IQR: 1.4-4.6 years) and 2.8 years (IQR: 1.4-4.5 years), respectively. Unadjusted risks for OS and DFS demonstrated significant differences across CALLY index category (both P < 0.001) (**Figures 1A, B**).

### Association between CALLY index and OS/DFS

Patients with CALLY index > 2.55 were significantly associated with a prolonged OS than those patients with CALLY index ≤ 2.55, independent of established risk factors (main adjusted HR: 0.55; 95% CI: 0.43-0.71). The significance was also observed in change 1 unit in CALLY index (main adjusted HR: 0.96; 95% CI: 0.95-0.98) as well as 1 SD in CALLY index (main adjusted HR: 0.67; 95% CI: 0.54-0.82). Accordingly, DFS in the CALLY index > 2.55 group was significantly prolonged than CALLY index≤ 2.55 group (HR: 0.51; 95% CI: 0.40-0.65; increase 1 unit [HR: 0.96; 95% CI: 0.94-0.98] and 1 SD [HR: 0.65; 95% CI: 0.53-0.80] in CALLY index). Additional adjustment for surgical information did not attenuate the association (**Table 2**).

**Table 2:** The association between CALLY and risk of OS and DFS in the Shanghai General Hospital patient cohort (training cohort).

	Events/Patients	Event Rate Events/100PY	Unadjusted, HR (95% CI)	Adjusted for Age and Sex, HR (95% CI)	Main Adjustment, HR^a^ (95% CI)	Additional Adjustment, HR^b^ (95% CI)
OS						
Per 1 unit	277/553	17.88	0.96 (0.94-0.98)	0.96 (0.94-0.98)	0.96 (0.95-0.98)	0.97 (0.95-0.98)
Per 1 SD	277/553	17.88	0.62 (0.50-0.77)	0.64 (0.52-0.79)	0.67 (0.54-0.82)	0.66 (0.53-0.81)
CALLY ≤ 2.55	162/257	24.74	Ref	Ref	Ref	Ref
CALLY > 2.55	115/296	12.85	0.51 (0.40-0.65)	0.53 (0.42-0.67)	0.55 (0.43-0.71)	0.56 (0.44-0.72)
DFS						
Per 1 unit	302/553	19.71	0.96 (0.94-0.98)	0.96 (0.94-0.97)	0.96 (0.94-0.98)	0.96 (0.95-0.98)
Per 1 SD	302/553	19.71	0.61 (0.50-0.75)	0.63 (0.51-0.78)	0.65 (0.53-0.80)	0.64 (0.52-0.78)
CALLY ≤ 2.55	180/257	27.96	Ref	Ref	Ref	Ref
CALLY > 2.55	122/296	13.73	0.48 (0.38-0.61)	0.49 (0.39-0.63)	0.51 (0.40-0.65)	0.52 (0.41-0.65)

Hazard ratios (95% confidence interval) for the association between CALLY and OS, DFS.

a Adjusted for age, sex, body mass index, hypertension, diabetes mellitus, cerebrovascular accident, tumor location, histological type, degree of differentiation, and TNM stage.

b Main adjustment + surgical information (duration of surgery, anastomosis site, intra-operative red blood cell transfusion, and intra-operative blood loss).

OS, overall survival; DFS, disease-free survival; PY, person-years; HR, hazard ratios; CI, confidence interval; Ref, reference; CALLY, C-reactive protein-albumin-lymphocyte index.

### Subgroup analysis

**Figure 2:**
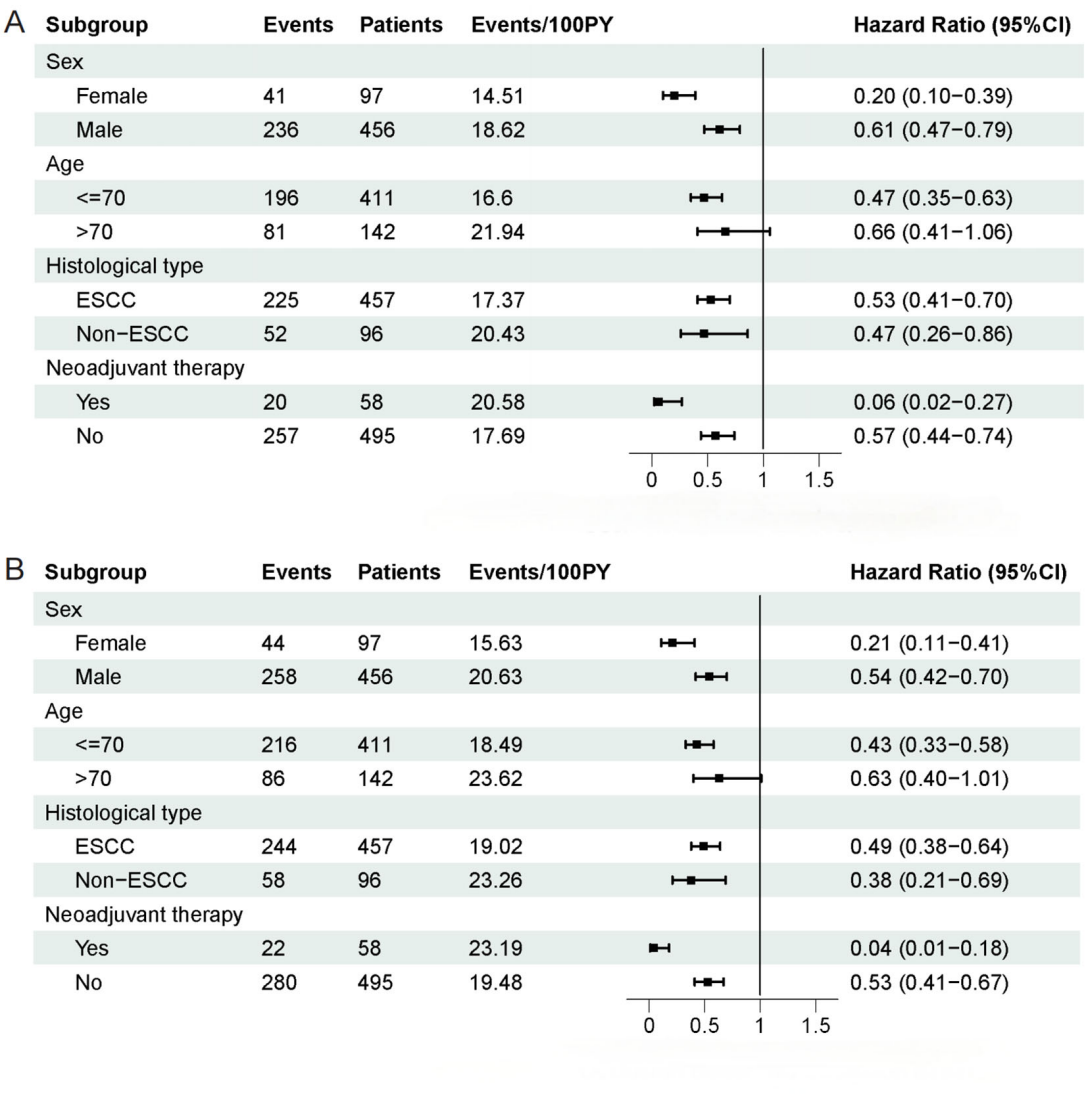
Subgroup analysis of overall survival and disease-free survival in patients. **(A)** Subgroup analysis of overall survival in patients. **(B)** Subgroup analysis of disease-free survival in patients.

Subgroup analysis was carried out according to sex (male/female), age (≤ 70 years/> 70 years), histological type (ESCC/non-ESCC), and neoadjuvant therapy (yes/no). Except for the subgroup of patients aged > 70 years, which did not show a statistically significant difference, the results of the subgroup analysis were in line with the overall analysis, indicating that the effect of CALLY index was consistent across different clinical conditions (**Figures 2A, B**).

### Influence of interim AC

An interim AC was observed in 101 patients (18.3%), of whom 62 (61.4%) died, and 74 (73.3%) developed non-DFS events during follow-up. Higher CALLY index was independently associated with a decreased risk of interim AC (**Supplementary Table S3**). Patients with AC had a significantly shorter OS (HR: 1.80; 95% CI: 1.34-2.41) and DFS (HR: 1.49; 95% CI: 1.15-1.94, see **Supplementary Table S4**). However, the association between CALLY index and OS/DFS was not attenuated after adjusting for interim AC, suggesting that the impact of CALLY index on long-term prognosis is independent of AC (**Supplementary Table S5**).

### Reverse causality analysis

Repeating the analyses after excluding patients who had event of death or tumor recurrence within the first 0.5, 1, and 2 years after inclusion yielded almost identical results. The association between CALLY index and OS/DFS was consistent over time, and higher baseline level of CALLY index remained significantly associated with prolonged OS and DFS even beyond 2 years after the initial measurement (**Supplementary Table S6**). The association between CALLY index and OS/DFS was not significantly modified by any of the prespecified clinical variables.

### Combined effect of CALLY index and established risk factors

Next, we constructed clinical prediction models for predicting OS and DFS based on established risk factors (ie, age, gender, BMI, hypertension, DM, neoadjuvant chemotherapy, radiotherapy, histological type, differentiation degree, TNM stage, and Ki67). Multicollinearity of the variables was evaluated and excluded by calculating variance inflation factor. The maximum VIF was 1.25, indicating that no multicollinearity exists between variables. The AUROC of the clinical risk models for predicting OS and DFS were 0.719 (95% CI: 0.677-0.761) and 0.745 (95% CI: 0.703-0.786), respectively. Adding CALLY index (continuous or categorical variable) into the models significantly improve its discriminative power for OS and DFS (all P < 0.01, **Supplementary Figure S3**, **Supplementary Table S7**).

### Comparison of CALLY index with NLR, PLR, and IINS

When combined with the clinical risk model, the CALLY index demonstrated superior prognostic performance for both OS and DFS compared to NLR, PLR, and IINS. For OS, the AUROC of the clinical risk model increased from 0.719 (95% CI: 0.677-0.761) to 0.752 (95% CI: 0.712-0.793) when CALLY index was added, which was significantly higher than the improvements observed with IINS (0.737, 95% CI: 0.696-0.779), NLR (0.718, 95% CI: 0.676-0.761), and PLR (0.721, 95% CI: 0.678-0.763; all P < 0.01). Similarly, for DFS, the AUROC of the clinical risk model increased from 0.745 (95% CI: 0.703-0.786) to 0.788 (95% CI: 0.750-0.825) with the addition of CALLY index, outperforming IINS (0.767, 95% CI: 0.727-0.806), NLR (0.745, 95% CI: 0.704-0.786), and PLR (0.747, 95% CI: 0.706-0.788; all P < 0.01) (**Supplementary Table S8**, **Supplementary Figure S4**). These results indicate that the CALLY index provides superior prognostic value compared to NLR, PLR, and IINS.

### Association between CALLY and recurrence in competing risk model

The association between CALLY index and recurrence was compared in the Fine and Gray model. The cumulative recurrence rate in the CALLY index > 2.55 group was significantly lower than that in the CALLY index ≤ 2.55 group (main adjusted HR, 0.51; 95%CI, 0.39-0.67) (**Supplementary Table S9**, **Supplementary Figure S5**). The difference was also significant in change 1 unit (main adjusted HR: 0.95; 95% CI: 0.93-0.98) and 1 SD (main adjusted HR: 0.60; 95% CI: 0.45-0.79) in CALLY index, as well as the model with additional adjustments.

### Validation of CALLY in the SHSH patient cohort

**Figure 3:**
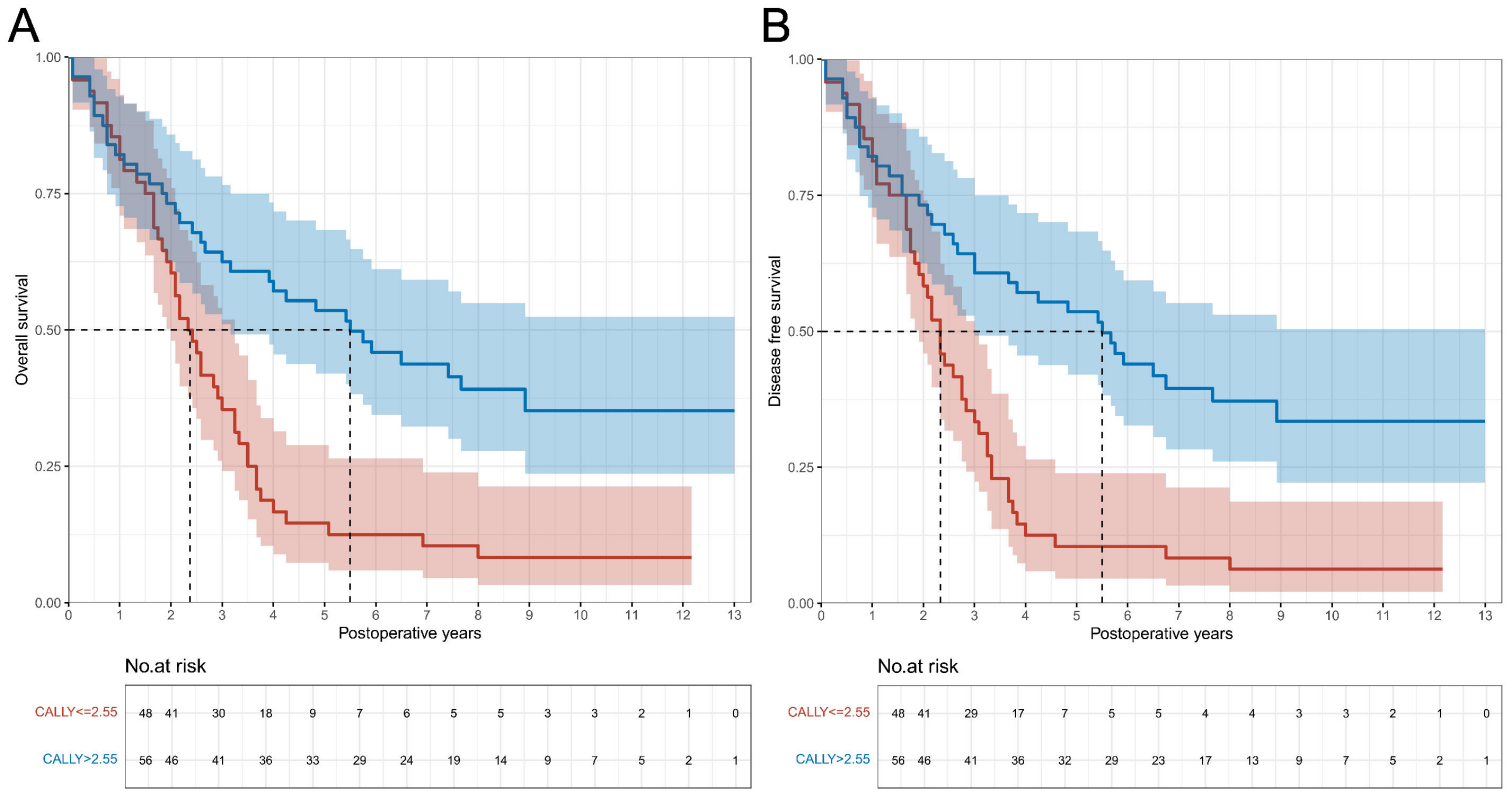
Overall survival and disease-free survival curves in patients with CALLY index ≤ 2.55 vs. CALLY index > 2.55 in the validation cohort. **(A)** Comparison of overall survival curves between patients with CALLY index ≤ 2.55 and CALLY index > 2.55. **(B)** Comparison of disease-free survival curves between patients with CALLY index ≤ 2.55 and CALLY index > 2.55. CALLY, C-reactive protein-albumin-lymphocyte.

From November 2010 to December 2018, a total of 104 patients with esophageal cancer were enrolled in SHSH (with a median follow-up of 3.1 years [IQR: 1.7-6.6 years]). Baseline clinical characteristics and in-hospital outcomes of the cohort are shown in **Supplementary Table S10**. The same CALLY threshold was applied to validate our results from the training cohort. **Figures 3A, B** show survival curves stratified by the CALLY index. Patients with CALLY index > 2.55 had significantly better OS and DFS compared to those with CALLY index ≤ 2.55 (both P < 0.001). After multivariable adjustment, a high CALLY index level was significantly associated with a decreased risk of poor OS and DFS (**Supplementary Table S11**). Similarly, in the Fine and Gray model, patients with CALLY index > 2.55 showed a significantly reduced risk of recurrence (**Supplementary Table S12**, **Supplementary Figure S6**).

## Discussion

In this study, we conducted a multi-center, retrospective cohort specifically for EC patients to evaluate the prognostic value of the CALLY index in mid- to long-term outcomes following esophagectomy. Our results demonstrate that patients with a CALLY index exceeding 2.55 exhibit significantly prolonged OS and DFS compared to those with a CALLY index of 2.55 or below. Additionally, the risk of recurrence was markedly reduced in the higher CALLY index group. This association retained its independent prognostic significance even after adjusting for potential confounders, including demographic characteristics, tumor staging, and surgical details. What is more, the CALLY index significantly enhances the discriminatory power of well-established clinical risk models for both OS and DFS, and does so better than NLR, PLR and IINS. These findings suggest that the CALLY index could serve as a reliable indicator for improving prognostic stratification in patients with EC.

The prognostic utility of the CALLY index likely arises from its ability to comprehensively evaluate systemic inflammation, nutritional status, and immune function. As an integrated metric, CALLY index reflects systemic inflammatory responses; specifically, high levels of CRP lead to low CALLY index values, indicating heightened systemic inflammation. Both acute and chronic inflammation have been extensively validated as being closely associated with tumor progression and poor prognosis (23–25). The CRP component of CALLY index is an acute-phase protein synthesized by hepatocytes or cancer cells, which can create a favorable environment for tumor growth, induce DNA damage, promote angiogenesis, and facilitate tumor dissemination and metastasis, thereby indicating the level of inflammation within the body (26, 27). Additionally, a low CALLY index is linked to the deterioration of the patient’s nutritional status. Preoperative nutritional status is a significant factor influencing postoperative complications and long-term survival rates (28), with serum albumin serving as a direct indicator of nutritional level. Hypoalbuminemia directly reflects a patient’s malnutrition status; albumin is crucial for maintaining colloid osmotic pressure and supporting tissue repair. A decrease in albumin levels can lead to tissue edema, thereby compromising blood supply to the anastomotic site and increasing the risk of ischemic leaks due to reduced perfusion and oxygenation at the healing site. Moreover, poor nutritional status compromises mucosal barrier integrity, increasing the risk of opportunistic infections, including bacterial translocation. Lastly, lymphocyte count depletion may reflect impaired immune surveillance, thereby weakening the body’s ability to monitor tumors. Furthermore, inflammatory factors may also suppress immune function, creating a reciprocal interaction (29). These interconnected mechanisms collectively underscore the clinical utility of CALLY index as a comprehensive prognostic tool for esophageal cancer.

AC, especially anastomotic leaks, represent some of the most frequent and serious adverse events after esophagectomy. Such complications not only prolong hospital stays and escalate healthcare costs but also increase both short- and long-term mortality rates (30). According to a study by Hao Xu et al., preoperative low albumin levels were identified as a significant risk factor for anastomotic leaks based on an analysis of data from 382 patients (31). CRP is an acute-phase reactant whose concentration significantly rises during inflammation, infection, or tissue damage. In cases of acute infection and inflammatory edema, tissues become fragile, and the healing process is delayed, substantially increasing the risk of leaks. Additionally, chronic inflammatory stimuli may lead to tissue fibrosis, subsequently resulting in anastomotic stricture (32). Moreover, lymphocytes, as key components of the immune system, play a vital role in defending against pathogen invasion, regulating immune responses, and maintaining immune homeostasis. Studies have shown that patients with lower lymphocyte counts are more susceptible to infections compared to those with higher absolute lymphocyte count (33). This study delves into the relationship between the CALLY index and AC, revealing that patients with higher CALLY index indices face a relatively lower risk of such complications. After further adjusting for AC in the well-established risk model and conducting mediated effects analysis, the association between CALLY and OS/DFS remained unchanged, indicating that CALLY influences patients’ long-term prognosis through pathways independent of AC. Additionally, a reverse causality analysis yielded results highly consistent with prior findings, confirming the unidirectional and unequivocal association of the CALLY index with OS and DFS. This effect persisted unaffected by subsequent treatment.

In the subgroup analysis of EC patients undergoing neoadjuvant therapy, the CALLY index demonstrated significantly enhanced predictive efficacy for OS (HR: 0.06 vs. 0.57) and DFS (HR: 0.04 vs. 0.53) indicating that neoadjuvant therapy amplifies the risk stratification and prognostic capabilities of CALLY index. In other words, patients undergoing neoadjuvant therapy with low CALLY index levels are more likely to have a poorer expected survival after surgery. Neoadjuvant therapy may improve the prognosis of patients with low CALLY index values through various mechanisms, including the modulation of immune function to alleviate immunosuppressive states, reducing tumor staging to decrease tumor burden, improving nutritional status, and lowering systemic inflammatory factor levels, ultimately leading to improved clinical outcomes. Conversely, low CALLY index levels after neoadjuvant therapy may indicate a poor response to treatment or that the patient’s baseline condition is already quite unfavorable.

The addition of the CALLY index to the well-established clinical risk model significantly enhances its predictive capability for OS and DFS in EC patients following esophagectomy. Compared to the NLR, PLR, and IINS, the CALLY index demonstrates superior predictive performance. This advantage likely stems from its multidimensional design, which integrates CRP, albumin, and lymphocyte counts to provide a more comprehensive assessment of systemic inflammation, nutritional status, and immune function. In contrast, NLR and PLR focus solely on single dimensions of inflammation and immunity, failing to capture the patient’s overall health status comprehensively. Although IINS incorporates CRP, lymphocyte count, and albumin, its stratified scoring method-dividing each indicator into grades 1, 2, and 3 before summation-may inadequately reflect the continuous variations of these indicators and their impact on prognosis. This discretized scoring approach may result in information loss, thereby limiting its predictive efficacy.

While the TNM staging system remains the gold standard for predicting postoperative oncological outcomes, its predictive accuracy is often limited due to the increasing complexity of prognostic factors and the diversification of treatment modalities (5). In this study, the CALLY index serves as a novel prognostic biomarker that effectively complements traditional TNM staging in risk stratification. When CALLY index was incorporated into the well-established clinical prediction model that included TNM staging, the model’s discriminatory ability for OS and DFS significantly improved (AUC increased from 0.719 and 0.745 to 0.752 and 0.788, respectively), indicating that CALLY index can identify high-risk populations that traditional risk factors fail to differentiate. These findings suggest that CALLY index can serve not only as an independent prognostic marker but also as a tool for patient risk stratification, guiding individualized treatment strategies. For high-risk patients (CALLY index ≤ 2.55), intensified perioperative interventions, such as preoperative nutritional support (e.g., enteral nutrition, albumin infusion) and anti-inflammatory therapies (e.g., non-steroidal anti-inflammatory drugs), should be considered to improve their baseline conditions (e.g., immune modulators).

Our findings hold substantial clinical implications. For EC patients with TNM stage II-III and CALLY index ≤ 2.55, neoadjuvant therapy is recommended to reduce tumor burden and improve the immune microenvironment. If CALLY index does not improve after neoadjuvant therapy, it may indicate a poor treatment response, necessitating timely adjustments to the treatment plan or suggesting a poor prognosis that requires careful consideration of further surgical intervention, potentially shifting to palliative care to reduce unnecessary healthcare resource consumption and patient burden. For low-risk patients (CALLY index > 2.55) with TNM stage I-II, a shorter postoperative adjuvant therapy duration may be considered to minimize treatment-related toxicity. Additionally, high CALLY index may indicate better baseline conditions and surgical tolerance, making these patients suitable candidates for minimally invasive surgical approaches (e.g., thoracoscopic or laparoscopic esophagectomy). In terms of postoperative complications, patients with CALLY index ≤ 2.55 face an increased risk of AC, suggesting the need for enhanced assessment of anastomotic blood supply during surgery and extended postoperative drainage tube placement. Furthermore, CALLY index can be utilized for patient stratification in clinical trials, reducing intergroup heterogeneity and improving the accuracy and reliability of efficacy assessments. Our study has several strengths. First, our study markedly improves the reliability and clinical relevance of its findings through methodological advancements and a robust multi-layered validation framework. Firstly, prior studies on the association between CALLY index and EC prognosis have often been single-center and small-sample investigations, with a singular approach to determining the cutoff values for CALLY index (34, 35). In contrast, this study is the largest known multi-center investigation to date. We applied a robust variable selection method to determine the optimal cutoff value for CALLY index, and thus avoiding biases from a single-method approach and enhancing the credibility of the results. Secondly, we constructed a survival prediction model for postoperative esophageal cancer incorporating well-known risk factors, demonstrating that adding this index robustly enhances the predictive value of the model, providing clinicians with a convenient and high-precision risk assessment tool to identify high-risk patients and guide accurate treatment strategies. Finally, we validated the effect of the CALLY index cutoff value on outcomes in a multi-center patient cohort, improving the generalizability of the results. Additionally, the follow-up analysis results from an independent external cohort were highly consistent with those of this study, confirming the robustness of the findings. In conclusion, our study not only addresses the limitations of previous studies, but also lays the groundwork for broader clinical applications and improved patient outcomes.

However, several limitations should also be considered. First, the retrospective nature of the cohort data, despite efforts to minimize confounding through a multi-center design and multivariable adjustments, leaves room for unmeasured variables—such as patient compliance and socioeconomic status-to influence prognostic outcomes. Prospective cohort studies are needed to validate the predictive efficacy of the CALLY index. Furthermore, the mechanisms linking CALLY index to prognosis, particularly inflammation-immune interactions, remain incompletely understood and warrant deeper exploration through experimental studies. Another limitation is the relatively low proportion of patients receiving neoadjuvant therapy (approximately 10.5%), which may introduce bias regarding its impact on the CALLY index. Additionally, the current median follow-up duration of 3.1 years may limit the assessment of long-term survival patterns; however, all patients remain actively monitored in our prospective follow-up program, with planned analyses comparing 5-year overall and recurrence-free survival between risk strata. Longitudinal studies focusing on neoadjuvant therapy are essential to assess dynamic changes in the CALLY index before and after treatment and their clinical implications. Despite these limitations, the core conclusions of this study remain robust. Moving forward, our research will aim to develop a CALLY-driven dynamic prognostic assessment system, enabling continuous risk stratification and timely, precise interventions to improve patient outcomes.

## Data Availability

The original contributions presented in the study are included in the article/**Supplementary Material**. Further inquiries can be directed to the corresponding authors.
