# Supplementary material for: Pretreatment CALLY index as promising novel biomarker in the prediction of surgical and oncological outcomes in esophageal cancer: a multi-center retrospective cohort study
Source: Front Immunol. 2025 May 21;16:1605067. doi: 10.3389/fimmu.2025.1605067 (PMC12133803; doi:10.3389/fimmu.2025.1605067)
Supplement: Supplementary file 1 [file DataSheet1.docx]

**Supplemental Material**

**Title: Pretreatment CALLY Index as Promising New Marker in the Prediction of Surgical and Oncological Outcomes in esophageal cancer**

Supplemental Method: Surgical approach p. 2

Supplemental Method: Statistical analysis p. 3

Supplemental Tables p. 5

Table S1. Intra-operative situation of patients with CALLY index ≤ 2.55 vs. CALLY index > 2.55 p. 5

Table S2. In-hospital outcomes of patients with CALLY index ≤ 2.55 vs. CALLY index > 2.55 p. 6

Table S3. Association between CALLY index and interim AC p. 7

Table S4. Association between interim AC and OS/DFS p. 8

Table S5. Association between CALLY index and OS/DFS adjusted for interim AC p. 9

Table S6. Reverse causality assessment p. 10

Table S7. Area under the receiver operating characteristic curve of clinical risk model p.11

combined with CALLY index

Table S8. Area under the receiver operating characteristic curve of biomarkers combined p.12

with clinical risk model

Table S9. Subdistribution hazard ratios for the association between CALLY index and incidence of p.13

recurrence in competing risk-adjusted models

Table S10. Perioperative clinical characteristics of patients with CALLY index ≤ 2.55 vs. CALLY index > 2.55 p.14

in the Shanghai Sixth People's Hospital patient cohort (validation cohort)

Table S11. The association between CALLY index and OS/DFS in the Shanghai Sixth People's Hospital p.16

patient cohort (validation cohort)

Table S12. Subdistribution hazard ratios for the association between CALLY index and incident p.17

recurrence in competing risk-adjusted models in the Shanghai Sixth People's Hospital

patient cohort (validation cohort)

Supplemental Figures p. 18

Figure S1. Flowchart of study enrollment p. 18

Figure S2. Density distribution of CALLY index in the training study population p. 19

Figure S3. The area under the curve of clinical risk models combined or not combined with p. 20

CALLY index in predicting OS and DFS

Figure S4. The area under the receiver operating characteristic curve of biomarkers combined p. 21

with clinical risk model

Figure S5. Cumulative incidence curves for the rate of recurrence in patients with p. 22

CALLY index ≤ 2.55 vs. CALLY index > 2.55

Figure S6. Cumulative incidence curve for the rate of recurrence in patients with p. 23

CALLY index ≤ 2.55 vs. CALLY index > 2.55 in the Shanghai Sixth People's Hospital

patient cohort (validation cohort)

Figure S7. Schoenfeld residuals plot of the CALLY index for OS/DFS over time p. 24

Supplemental References p. 25

**Surgical procedure for esophageal cancer**

(i) Sweet procedure is a type of transthoracic esophagectomy that involves a combined abdominal and thoracic approach. This procedure typically involves resecting the esophagus from the cervical or thoracic region and reconstructing it using the stomach. The stomach is mobilized, and the distal esophagus is removed. It starts with a laparotomy (abdominal incision) to mobilize the stomach. Then, a thoracotomy (incision in the chest) is made to access the esophagus. The esophagus is removed, and the stomach is used to form a new esophagus, which is anastomosed to the remaining esophagus or cervical esophagus. The Sweet procedure is often used in patients with tumors located in the middle or lower thirds of the esophagus. It's suitable for cases where the cancer involves the thoracic region but does not extend to the cervical or abdominal regions in a way that requires a more extensive resection(1); (ii) Ivor-Lewis Procedure: The Ivor-Lewis procedure is a two-stage surgery that combines both abdominal and right thoracic approaches. It is commonly used for esophageal cancer located in the distal esophagus or gastroesophageal junction. The surgery begins with a laparotomy to mobilize the stomach and prepare it for use as the conduit. The thoracic phase involves a right-sided thoracotomy to resect the lower esophagus. The stomach is then pulled up into the chest and connected to the remaining esophagus, creating a new pathway for food. It is typically indicated for cancers located in the distal esophagus or at the gastroesophageal junction. This procedure is often used when the tumor is localized to the lower esophagus or the junction between the esophagus and stomach(2); (iii) The McKeown procedure is a three-stage esophagectomy involving an abdominal, right thoracic, and cervical approach. It is more extensive and is typically performed when the cancer is located in the middle to upper esophagus. The first stage involves a laparotomy to mobilize the stomach. The second stage includes a right thoracotomy to remove the esophagus. The third stage involves a cervical incision to perform an anastomosis between the stomach and the remaining cervical esophagus, creating a new esophagus. The McKeown procedure is typically indicated for esophageal cancers located in the middle or upper portions of the esophagus, especially when the tumor is not resectable through a single thoracic or abdominal approach. This is a more complex surgery, often chosen for cancers with more extensive involvement of the esophagus(3).

**Statistical analysis**

Continuous variables are presented as means (SD) when normally distributed or as medians (IQR) for variables with skewed distribution. Categorical data are presented as percentages. Statistical differences between continuous or categorical variables were established using a Student’s t test, Mann Whitney U test, chi-square test, or Fisher‘s exact probability method as appropriate. Statistical analysis was performed using R (version 4.0.3). The R package used for analysis will be described in the corresponding paragraph. A two-sided P value < 0.05 was considered statistically significant.

As most of the variables were collected through manual review of electronic health records (EHR), missing data was unavoidable. Nevertheless, our dataset is highly complete, with missing data ranging from 0% to 2.6%. Multiple imputation methods were utilized to address the missing data. For continuous variables, predictive mean matching was employed for imputation. For binary variables, logistic regression was applied, while unordered categorical variables were imputed using unordered multinomial logistic regression. Ordered categorical variables were imputed using the proportional odds model. All imputations were performed using the R (version, 3.5.0), corresponding to "pmm", "logreg", "polyreg", and "polr" method in the packages of *mice* (version 3.13.0). A total of five imputations were conducted for all missing values. The final dataset used for analysis was derived from the extraction of data across the five imputed datasets.

To enhance the general readability of the manuscript, we established the association between CALLY index levels and overall survival (OS)/disease-free survival (DFS) using multivariable-adjusted Cox regression analyses, including CALLY index as a continuous or categorical variable dichotomized at 2.55. This cutoff value has been chosen based on three different cut point selection methods (Maximizing the Youden Index, Decision Survival Tree, and Non-restricted Spline Regression). (i) Maximizing the Youden Index: To determine the optimal cutoff value, we plotted the area under the receiver operating characteristic curve (AUROC) curve for CALLY index values against OS. The Youden index was maximized by calculating the sum of sensitivity and specificity, then subtracting 1. This cutoff corresponds to the point where the diagnostic performance of the model is optimized; (ii) Decision Survival Tree: The key idea behind determining the cutoff value in a decision survival tree is to iteratively evaluate different potential split points, selecting those that maximize the difference in survival outcomes. For each candidate cutoff, the survival differences between the high-risk and low-risk groups after the split are computed; (iii) Non-restricted Spline Regression: Non-restricted spline regression models non-linear relationships in the data by dividing it into segments and fitting a separate polynomial function to each segment. The cutoff value is typically determined based on the data distribution or the optimal model fit, with the best segment boundaries selected through methods such as cross-validation. Then using the backward stepwise regression method, we constructed three Cox models for predicting OS including CALLY index dichotomized at three different cutoffs. Based on the lowest Akaike information criterion and Bayesian information criterion, we selected an optimal cutoff for CALLY index of 2.55 for subsequent analyses.

Cox proportional hazards models for the association between CALLY index and OS were derived in all patients with CALLY index ≤ 2.55 (n = 257). Models were progressively adjusted for potential confounders. First, the model was adjusted for age and sex. the model was adjusted for body mass index (BMI), hypertension, diabetes mellitus (DM), cerebrovascular accident, neoadjuvant chemotherapy, and tumor characteristics (ie, tumor location, histological type, differetiation, and TNM stage). In an exploratory model, additional adjustment for surgery information (duration of surgery, anastomosis site, intraoperative-red blood cell transfusion, and intra-operative blood loss) in the cohort was performed. We identified these potential factors based on clinical experience, statistical considerations, and previous literature reports. The association between CALLY index and OS/DFS was described using adjusted hazard ratios (HRs) for every 1 unit, 1 SD increase in CALLY index, and for CALLY index classification (CALLY index ≤ 2.55 vs. CALLY index > 2.55). Survival analysis was performed using *survival* (version 3.2-7) and *survminer* (version 0.5.0) packages. The Cox analysis was performed following the proportional hazard assumption. The proportional hazard assumption, assessed based on scaled Schoenfeld residuals and plots of the CALLY index for OS/DFS over time, was not violated for any of the models (Schoenfeld Individual Test P > 0.05 indicates that the relationship between the residuals and time is not significant, with no obvious time dependence, suggesting that the proportional hazards assumption is appropriate, **see Supplemental Figure S7**).

The association between CALLY index and recurrence was performed using competing risk analysis in the Fine and Gray models, by treating death from other causes as a competing risk. This means censoring was applied at the time of unrelated death (i.e. death not preceded by tumor factor), or at the end of follow-up. The influence of the recurrence (not preceded by death) was assessed by deriving subdistribution HRs (SHRs) from the Fine and Gray models. In line with the primary analysis, SHRs from Fine and Gray models (including the same sets of potential confounders) were derived for every 1 unit, and 1 SD increase in CALLY index, and for CALLY index classification. Competing risk analysis was performed using the *cmprsk* package (2.2-10).

A subgroup analysis was performed to explore the differences in OS and DFS across various demographic and tumor characteristics. The patients were divided into subgroups based on age (≤ 70 years vs. > 70 years), gender (male vs. female), histological type (esophageal squamous cell carcinoma [ESCC] vs. non-ESCC), and neoadjuvant therapy (yes vs. no). Additionally, an interaction effect analysis was conducted to assess the interplay between these variables.

Anastomotic complications (AC) in esophageal cancer are important factors that affecting patient prognosis. Next, we investigated whether the association between CALLY index and OS/DFS was mediated by the occurrence of AC, by adjusting the AC as a time-varying covariate. Anastomotic complications are defined as anastomosis fistula, marginal ulcer, and anastomotic stenosis that need dilation treatment or stent implantation.

Reverse causality was assessed by repeating the analyses after excluding patients with OS and DFS within the first 0.5, 1, and 2 years after inclusion. Consistency of the association between CALLY index and OS/DFS over time was assessed by determining the effects of CALLY index on events risk within subsequent time intervals.

In addition, we explored the additional predictive value of CALLY index by adding it to the clinical risk model. We constructed a Cox regression model for OS by incorporating age, gender, BMI, admission comorbidities (hypertension, DM, neoadjuvant chemotherapy, and radiotherapy), and TNM stage; and a Cox regression model for DFS by incorporating age, BMI, hypertension, DM, neoadjuvant chemotherapy, radiotherapy, histological type, differentiation, and TNM stage. Multicollinearity of the variables was evaluated and excluded by calculating variance inflation factor (VIF). The max VIF was 1.25, indicating no multicollinearity existed between variables. The area under the receiver operating characteristic curve (AUROC) was performed to evaluate the discrimination of the models. DeLong test, a non-parametric method based on standard error and covariance, was applied to compare the difference in AUC between two ROC curves.

**Table S1 Intra-operative situation of patients with CALLY ≤ 2.55 vs. CALLY > 2.55**

| **Intra-operative variable** | **CALLY index ≤ 2.55**  **(n = 257)** | **CALLY index > 2.55**  **(n = 296)** | **P value** |
| --- | --- | --- | --- |
| Perineural invasion | 88 (34.2%) | 108 (36.5%) | 0.645 |
| Vessel invasion | 93 (36.2%) | 92 (31.1%) | 0.238 |
| esophageal resection margin (+) | 9 (3.5%) | 10 (3.4%) | 1.000 |
| Gastric resection margin (+) | 1 (0.4%) | 2 (0.7%) | 1.000 |
| Surgical duration | 326.4 (112.4) | 314.7 (98.5) | 0.197 |
| Jejunostomy | 142 (55.3%) | 162 (54.7%) | 0.970 |
| Other surgeries | 32 (12.5%) | 37 (12.5%) | 1.000 |
| Video-assisted thoracoscopic surgery | 40 (15.6%) | 69 (23.3%) | 0.029 |
| Laparoscopic surgery | 28 (10.9%) | 33 (11.1%) | 1.000 |
| Intraoperative red blood cell transfusion | 16 (6.2%) | 4 (1.4%) | 0.005 |
| Intraoperative blood plasma transfusion | 15 (5.8%) | 3 (1.0%) | 0.003 |
| Intraoperative blood loss | 200.0 (100.0, 360.0) | 200.0 (100.0, 342.5) | 0.129 |

Data are presented as medians (IQR) and percentages.

Abbreviations: CALLY index, C-reactive protein-albumin-lymphocyte index;IQR, Interquartile Range.

**Table S2 In-hospital outcomes of patients with CALLY index ≤ 2.55 vs. CALLY index > 2.55**

| **In-hospital Outcome** | **CALLY index ≤ 2.55**  **(n = 257)** | **CALLY index > 2.55**  **(n = 296)** | **P value** |
| --- | --- | --- | --- |
| Post-operative red blood cell transfusion | 28 (10.9%) | 38 (12.8%) | 0.568 |
| Acute kidney injury^a^ | 9 (3.5%) | 7 (2.4%) | 0.426 |
| Hepatic insufficiency^b^ | 18 (7.0%) | 28 (9.5%) | 0.297 |
| In-hospital death | 10 (3.9%) | 8 (2.7%) | 0.432 |

^a^ Acute kidney injury is defined according to the Scrbased criteria from the Kidney Disease: Improving Global Outcomes (KDIGO) consensus definition, specifically an acute increase in Scr ≥ 50% within 7 days or ≥ 0.3 mg/dL within 48 h compared with the baseline level, or a requirement for renal replacement therapy.

^b^ Hepatic insufficiency is an increase of alanine aminotransferase (≥80 U/L) and bilirubin (≥12 mg/dL) in blood, with abnormal function of synthesis and metabolism.

Abbreviations: CALLY index, C-reactive protein-albumin-lymphocyte index.

**Table S3. Association between CALLY index and interim AC**

|  | **Events/Patients** | **Event rate (events/ 100 PY)** | **Unadjusted,**  **HR (95% CI)** | **Adjusted for age and sex,**  **HR (95% CI)** | **Main adjustment^a^,**  **HR (95% CI)** | **Additional adjustment^b^,**  **HR (95% CI)** |
| --- | --- | --- | --- | --- | --- | --- |
| Per 1 unit | 101/553 | 6.55 | 0.93 (0.89-0.96) | 0.94 (0.89-0.97) | 0.93 (0.90-0.97) | 0.93  (0.89-0.97) |
| Per 1 SD | 101/553 | 6.55 | 0.42 (0.26-0.69) | 0.45 (0.29-0.73) | 0.45 (0.28-0.72) | 0.44  (0.27-0.71) |
| CALLY index ≤ 2.55 | 57/257 | 8.77 | Ref | Ref | Ref | Ref |
| CALLY index > 2.55 | 44/296 | 4.94 | 0.56 (0.38-0.84) | 0.60 (0.40-0.90) | 0.61 (0.41-0.93) | 0.62  (0.41-0.94) |

Hazard ratios (95% confidence interval) for the association between CALLY index and interim AC.

^a^ Adjusted for age, sex, body mass index, hypertension, diabetes mellitus, cerebrovascular accident, tumor location, histological type, degree of differentiation, and TNM stage

^b^ Main adjustment + surgical information (duration of surgery, anastomosis site, intra-operative red blood cell transfusion, and intra-operative blood loss).

Abbreviations: CALLY index, C-reactive protein-albumin-lymphocyte index; AC, anastomotic complications; PY, person-years; HR, hazard ratios; CI, confidence interval; SD, standard deviation; Ref, reference.

**Table S4. Association between interim AC and OS/DFS**

|  | **Events/**  **Patients** | **Event rate (events/ 100 PY)** | **Unadjusted,**  **HR (95% CI)** | **Adjusted for age and sex,**  **HR (95% CI)** | **Main adjustment^a^,**  **HR (95% CI)** | **Additional adjustment^b^,**  **HR (95% CI)** |
| --- | --- | --- | --- | --- | --- | --- |
| **Anastomotic complication** | | | | | | |
| **OS** | | | | | | |
| No^c^ | 215/553 | 13.96 | Ref | Ref | Ref | Ref |
| Yes | 62/101 | 25.93 | 1.81 (1.36-2.40) | 1.76 (1.32-2.34) | 1.80 (1.34-2.41) | 1.84 (1.38-2.47) |
| **DFS** | | | | | | |
| No^c^ | 228/553 | 14.88 | Ref | Ref | Ref | Ref |
| Yes | 74/101 | 30.82 | 1.56 (1.21-2.02) | 1.52 (1.17-1.96) | 1.49 (1.15-1.94) | 1.53 (1.18-1.99) |

Hazard ratios (95% CI) for the association between interim anastomotic complications and OS, DFS. Interim AC was analyzed as a time-varying covariate, i.e. patients who had an interim AC were analyzed in the group without an interim AC until they had the interim event, after which they were moved to the group with an interim AC for the remainder of follow-up.

^a^ Adjusted for age, sex, body mass index, hypertension, diabetes mellitus, cerebrovascular accident, tumor location, histological type, degree of differentiation, and TNM stage

^b^ Main adjustment + surgical information (duration of surgery, anastomosis site, intra-operative red blood cell transfusion, and intra-operative blood loss).

^c^ This group includes all patients (n = 553) because interim AC is analyzed as a time-varying covariate. At the start of follow-up no patient has an interim AC, and therefore all patients are first included in the group without an interim AC. As soon as patients have an interim AC, they are moved to the group with an interim AC and stay in this group for the remainder of follow-up.

Abbreviations: AC, anastomotic complications; OS, overall survival; DFS, disease-free survival; PY, person-years; HR, hazard ratios; CI, confidence interval; Ref, reference.

**Table S5. Association between CALLY and OS/DFS adjusted for interim AC**

|  | **Events/**  **Patients** | **Event rate (events/ 100 PY)** | **Main adjustment^a^,**  **HR (95% CI)** | **Main adjustment^a^ + interim AC,**  **HR (95% CI)** | **Additional adjustment^b^,**  **HR (95% CI)** | **Additional adjustment^b^ + interim AC,**  **HR (95% CI)** |
| --- | --- | --- | --- | --- | --- | --- |
| **OS** | | | | | | |
| Per 1 unit | 277/553 | 17.88 | 0.96 (0.95-0.98) | 0.96 (0.96-0.99) | 0.97 (0.95-0.98) | 0.97 (0.96-0.98) |
| Per 1 SD | 277/553 | 17.88 | 0.67 (0.54-0.82) | 0.69 (0.55-0.85) | 0.66 (0.53-0.81) | 0.67 (0.54-0.83) |
| CALLY index ≤ 2.55 | 162/257 | 24.74 | Ref | Ref | Ref | Ref |
| CALLY index > 2.55 | 115/296 | 12.85 | 0.55 (0.43-0.71) | 0.58 (0.45-0.73) | 0.56 (0.44-0.72) | 0.58 (0.45-0.74) |
| **DFS** | | | | | | |
| Per 1 unit | 302/553 | 19.71 | 0.96 (0.94-0.98) | 0.97 (0.94-0.98) | 0.96 (0.95-0.98) | 0.97 (0.95-0.98) |
| Per 1 SD | 302/553 | 19.71 | 0.65 (0.53-0.80) | 0.68 (0.55-0.82) | 0.64 (0.52-0.78) | 0.65 (0.53-0.79) |
| CALLY index ≤ 2.55 | 180/257 | 27.96 | Ref | Ref | Ref | Ref |
| CALLY index > 2.55 | 122/296 | 13.73 | 0.51 (0.40-0.65) | 0.53 (0.42-0.68) | 0.52 (0.41-0.65) | 0.53 (0.41-0.66) |

Hazard ratios (95% CI) for the association between CALLY index and OS, DFS with and without adjustment for interim AC. Interim AC was analyzed as a time-varying covariate, i.e. patients who had an interim AC were analyzed in the group without an interim AC until they had the interim event, after which they were moved to the group with an interim AC for the remainder of follow-up.

^a^ Adjusted for age, sex, body mass index, hypertension, diabetes mellitus, cerebrovascular accident, tumor location, histological type, degree of differentiation, and TNM stage

^b^ Main adjustment + surgical information (duration of surgery, anastomosis site, intra-operative red blood cell transfusion, and intra-operative blood loss).

Abbreviations: CALLY index, C-reactive protein-albumin-lymphocyte index; OS, overall survival; DFS, disease-free survival; AC, anastomotic complications; PY, person-years; HR, hazard ratios; CI, confidence interval; SD, standard deviation; Ref, reference.

**Table S6. Reverse causality assessment**

|  | **Total population** | **Exclude events < 0.5 year** | **Exclude events < 1 year** | **Exclude events < 2 year** |
| --- | --- | --- | --- | --- |
| **OS** | | | | |
| Events/Patients | 277/553 | 241/517 | 198/448 | 139/337 |
| HR (95% CI)  per 1 unit CALLY index | 0.96 (0.95-0.98) | 0.97 (0.95-0.98) | 0.97 (0.95-0.99) | 0.96 (0.94-0.99) |
| HR (95% CI)  per SD CALLY index | 0.67 (0.54-0.82) | 0.71 (0.57-0.87) | 0.70 (0.55-0.88) | 0.68 (0.51-0.90) |
| HR (95% CI)  CALLY index ≤ 2.55 | Ref | Ref | Ref | Ref |
| HR (95% CI)  CALLY index > 2.55 | 0.55 (0.43-0.71) | 0.54 (0.42-0.71) | 0.54 (0.40-0.72) | 0.56 (0.40-0.80) |
| **DFS** | | | | |
| Events/Patients | 302/553 | 265/516 | 216/442 | 156/333 |
| HR (95% CI)  per 1 unit CALLY index | 0.96 (0.94-0.98) | 0.96 (0.95-0.98) | 0.96 (0.95-0.98) | 0.96 (0.94-0.99) |
| HR (95% CI)  per 1 SD CALLY index | 0.65 (0.53-0.80) | 0.68 (0.55-0.83) | 0.67 (0.53-0.84) | 0.65 (0.49-0.87) |
| HR (95% CI)  CALLY index ≤ 2.55 | Ref | Ref | Ref | Ref |
| HR (95% CI)  CALLY index > 2.55 | 0.51 (0.40-0.65) | 0.49 (0.38-0.63) | 0.49 (0.37-0.64) | 0.51 (0.37-0.72) |

Hazard ratios per 1 mg/L and per SD CALLY index in the total population, and after excluding patients who had death from all causes within the first 0.5, 1, and 2 years after inclusion. Hazard ratios were adjusted for age, sex, body mass index, hypertension, diabetes mellitus, cerebrovascular accident, tumor location, histological type, degree of differentiation, and TNM stage.

Abbreviations: CALLY index, C-reactive protein-albumin-lymphocyte index; OS, overall survival; DFS, disease-free survival; HR, hazard ratios; CI, confidence interval; SD, standard deviation; Ref, reference.

**Table S7. Area under the receiver operating characteristic curve of clinical risk model combined with CALLY index**

| **AUROC** | **Clinical risk model^a^** | **Clinical risk model + CALLY index (continuous)** | **Clinical risk model + CALLY index (categorical)** | **P value^b^** | **P value^c^** |
| --- | --- | --- | --- | --- | --- |
| OS | 0.719 (0.677-0.761) | 0.752 (0.712-0.793) | 0.747 (0.707-0.788) | 0.002 | 0.01 |
| DFS | 0.745 (0.703-0.786) | 0.788 (0.750-0.825) | 0.782 (0.743-0.820) | <0.001 | <0.001 |

^a^ Clinical risk model is constructed using COX regression method. For predicting OS, the model includes age, gender, BMI, hypertension, diabetes mellitus, neoadjuvant chemotherapy, and radiotherapy, and TNM stage; for predicting DFS, the model includes age, BMI, hypertension, DM, neoadjuvant chemotherapy, radiotherapy, TNM stage, tumor type, and degree of differentiation.

^b^ Comparison of AUROC between clinical risk model and clinical risk model + CALLY index (continuous).

^c^ Comparison of AUROC between clinical risk model and clinical risk model + CALLY index (categorical).

Abbreviations: CALLY index, C-reactive protein-albumin-lymphocyte index; AUROC, area under the receiver operating characteristic curve; OS, overall survival; DFS, disease-free survival; HR, hazard ratios; CI, confidence interval; SD, standard deviation; Ref, reference.

**Table S8. Area under the receiver operating characteristic curve of biomarkers combined with clinical risk model**

| **AUROC** | **Clinical risk model^a^** | **Clinical risk model + CALLY index** | **Clinical risk model + IINS** | **Clinical risk model + NLR** | **Clinical risk model + PLR** |
| --- | --- | --- | --- | --- | --- |
| OS | 0.719 (0.677-0.761) | 0.752 (0.712-0.793) | 0.737 (0.696-0.779) | 0.718 (0.676-0.761) | 0.721 (0.678-0.763) |
| DFS | 0.745 (0.703-0.786) | 0.788 (0.750-0.825) | 0.767 (0.727-0.806) | 0.745 (0.704-0.786) | 0.747 (0.706-0.788) |

^a^ Clinical risk model is constructed using COX regression method. For predicting OS, the model includes age, gender, BMI, hypertension, diabetes mellitus, neoadjuvant chemotherapy, and radiotherapy, and TNM stage; for predicting DFS, the model includes age, BMI, hypertension, DM, neoadjuvant chemotherapy, radiotherapy, TNM stage, tumor type, and degree of differentiation.

Abbreviations: CALLY index, C-reactive protein-albumin-lymphocyte index; AUROC, area under the receiver operating characteristic curve; OS, overall survival; DFS, disease-free survival; CALLY, C-reactive protein-albumin-lymphocyte index; IINS, Immune-inflammatory-nutritional score; NLR, Neutrophil-to-lymphocyte ratio; PLR, Platelet-to-lymphocyte ratio.

**Table S9. Subdistribution hazard ratios for the association between CALLY and incident recurrence in competing risk-adjusted models**

|  | **Recurrence event** | **Competing events^a^** | **Unadjusted,**  **SHR (95% CI)** | **Adjusted for age and sex,**  **SHR (95% CI)** | **Main adjustment^a^,**  **SHR (95% CI)** | **Additional adjustment^b^,**  **SHR (95% CI)** |
| --- | --- | --- | --- | --- | --- | --- |
| Per 1 unit | 279 | 23 | 0.95 (0.93-0.97) | 0.95 (0.93-0.97) | 0.95 (0.93-0.98) | 0.95 (0.93-0.98) |
| Per 1 SD | 279 | 23 | 0.56 (0.42-0.74) | 0.58 (0.44-0.76) | 0.60 (0.45-0.79) | 0.58 (0.45-0.75) |
| CALLY index ≤ 2.55 | 169 | 11 | Ref | Ref | Ref | Ref |
| CALLY index > 2.55 | 110 | 12 | 0.48 (0.38-0.61) | 0.50 (0.39-0.64) | 0.51 (0.39-0.67) | 0.51 (0.40-0.65) |

Subdistribution hazard ratios (95% CI) for the association between CALLY index and recurrence. Analyses are performed in the Fine and Gray model.

^a^ Adjusted for age, sex, body mass index, hypertension, diabetes mellitus, cerebrovascular accident, tumor location, histological type, degree of differentiation, and TNM stage

^b^ Main adjustment + surgical information (duration of surgery, anastomosis site, intra-operative red blood cell transfusion, and intra-operative blood loss).

Abbreviations: CALLY, C-reactive protein-albumin-lymphocyte index; SHR, subdistribution hazard ratios; CI, confidence interval; SD, standard deviation; Ref, reference.

**Table S10. Perioperative clinical characteristics of patients with CALLY index ≤ 2.55 vs. CALLY index > 2.55 in the Shanghai Sixth People's Hospital patient cohort (validation cohort)**

| **Variable** | **CALLY index ≤ 2.55**  **(n=48)** | **CALLY index > 2.55**  **(n=56)** | **P value** |
| --- | --- | --- | --- |
| Age | 59.3±9.0 | 60.9±7.7 | 0.341 |
| Male | 37 (77.1%) | 48 (85.7%) | 0.378 |
| Body mass index | 21.8±4.0 | 21.9±3.4 | 0.896 |
| Hypertension | 2 (4.2%) | 13 (23.2%) | 0.013 |
| Diabetes mellitus | 1 (2.1%) | 7 (12.5%) | 0.069 |
| Cerebrovascular accident | 0 (0.0%) | 2 (3.6%) | 0.508 |
| Neoadjuvant chemotherapy | 1 (2.1%) | 1 (1.8%) | 1.000 |
| White blood cell count | 6.3 (4.7, 8.8) | 5.5 (4.7, 6.7) | 0.080 |
| Neutrophil count | 3.9 (2.9, 5.8) | 3.3 (2.7, 4.2) | 0.014 |
| Lymphocyte count | 1.0 (0.4, 1.5) | 1.6 (1.3, 1.8) | <0.001 |
| Mononuclear cell count | 0.5 (0.3, 0.6) | 0.4 (0.3, 0.6) | 0.355 |
| Red blood cell count | 4.3 (4.1, 4.6) | 4.6 (4.4, 4.9) | 0.007 |
| Hemoglobin | 136.5 (128.0, 144.0) | 143.5 (133.0, 150.8) | 0.045 |
| Platelet count | 197.0 (167.3, 228.0) | 197.0 (156.3, 237.5) | 0.660 |
| C-reactive protein | 22.0 (16.2, 29.1) | 8.6 (6.8, 15.6) | <0.001 |
| Serum creatinine | 74.0 (66.5, 85.0) | 69.5 (59.0, 77.0) | 0.016 |
| Blood glucose | 5.1 (4.7, 5.5) | 5.3 (4.9, 5.9) | 0.117 |
| Urea | 5.3 (4.5, 6.5) | 4.7 (4.1, 5.6) | 0.083 |
| Albumin | 40.5 (33.5,42.0) | 44.0 (40.0, 46.0) | <0.001 |
| Alanine aminotransferase | 14.5 (10.3, 26.0) | 16.0 (10.0, 23.8) | 0.539 |
| Total bilirubin | 9.9 (7.5, 15.7) | 11.7 (8.3, 13.9) | 0.224 |
| Tumor site |  |  | 0.615 |
| Thoracic | 46 (95.8%) | 55 (98.2%) |  |
| Abdomen | 2 (4.2%) | 1 (1.8%) |  |
| Degree of differentiation |  |  | 0.003 |
| Well | 45 (93.8%) | 40 (71.4%) |  |
| Moderate | 3 (6.2%) | 14 (25.0%) |  |
| Poor | 0 (0.0%) | 2 (3.6%) |  |
| TNM stage |  |  |  |
| T |  |  | 0.471 |
| T1 | 7 (14.6%) | 11 (19.6%) |  |
| T2 | 6 (12.5%) | 12 (21.4%) |  |
| T3 | 28 (58.3%) | 25 (44.6%) |  |
| T4 | 7 (14.6%) | 8 (14.3%) |  |
| N |  |  | 0.754 |
| N0 | 22 (45.8%) | 28 (50.0%) |  |
| N1 | 17 (35.4%) | 18 (32.1%) |  |
| N2 | 4 (8.3%) | 7 (12.5%) |  |
| N3 | 5 (10.4%) | 3 (5.4%) |  |
| Perineural invasion | 31 (64.6%) | 28 (50.0%) | 0.194 |
| Vessel invasion | 13 (27.1%) | 15 (26.8%) | 1.000 |
| esophageal resection margin (+) | 7 (14.6%) | 9 (16.1%) | 1.000 |
| Ki67 | 40.0 (30.0, 60.0) | 47.5 (30.0, 60.0) | 0.596 |
| Surgical duration | 310.0 (290.0, 350.0) | 320.0 (260.0, 350.0) | 0.421 |
| Intraoperative red blood cell transfusion | 10 (20.8%) | 9 (16.1%) | 0.710 |
| Intraoperative blood plasma transfusion | 8 (16.7%) | 5 (8.9%) | 0.372 |
| Postoperative red blood cell transfusion | 9 (18.8%) | 4 (7.1%) | 0.137 |
| Postoperative blood plasma transfusion | 26 (54.2%) | 36 (64.3%) | 0.396 |
| Intraoperative blood loss | 300.0 (100.0, 300.0) | 300.0(100.0, 300.0) | 0.730 |

Data are presented as means (SD), medians (IQR), or percentages.

Abbreviations: CALLY index, C-reactive protein-albumin-lymphocyte index.

**Table S11. The association between CALLY index and OS/DFS in the Shanghai Sixth People's Hospital patient cohort (validation cohort)**

|  | **Events/Patients** | **Event Rate**  **Events/100PY** | **Unadjusted, HR (95% CI)** | **Adjusted for Age and Sex, HR (95% CI)** | **Main Adjustment^a^, HR (95% CI)** | **Additional Adjustment^b^, HR (95% CI)** |
| --- | --- | --- | --- | --- | --- | --- |
| **OS** | | | | | | |
| Per 1 unit | 78/104 | 17.60 | 0.87 (0.82-0.93) | 0.87 (0.81-0.93) | 0.87 (0.81-0.94) | 0.88 (0.82-0.95) |
| Per 1 SD | 78/104 | 17.60 | 0.59 (0.45-0.77) | 0.57 (0.44-0.75) | 0.60 (0.45-0.79) | 0.62 (0.47-0.82) |
| CALLY index ≤ 2.55 | 44/48 | 29.30 | Ref | Ref | Ref | Ref |
| CALLY index > 2.55 | 34/56 | 11.61 | 0.42 (0.26-0.67) | 0.38 (0.24-0.61) | 0.39 (0.23-0.67) | 0.41 (0.24-0.71) |
| **DFS** | | | | | | |
| Per 1 unit | 80/104 | 18.56 | 0.87 (0.81-0.93) | 0.86 (0.81-0.92) | 0.88 (0.81-0.94) | 0.88 (0.82-0.95) |
| Per 1 SD | 80/104 | 18.56 | 0.59 (0.45-0.76) | 0.57 (0.44-0.74) | 0.60 (0.45-0.80) | 0.63 (0.47-0.83) |
| CALLY index ≤ 2.55 | 45/48 | 31.74 | Ref | Ref | Ref | Ref |
| CALLY index > 2.55 | 35/56 | 12.10 | 0.40 (0.25-0.63) | 0.36 (0.23-0.58) | 0.40 (0.23-0.68) | 0.42 (0.24-0.71) |

Hazard ratios (95% confidence interval) for the association between CALLY index and interim AC.

^a^ Adjusted for age, sex, body mass index, hypertension, diabetes mellitus, cerebrovascular accident, tumor location, histological type, degree of differentiation, and TNM stage

^b^ Main adjustment + surgical information (duration of surgery, anastomosis site, intra-operative red blood cell transfusion, and intra-operative blood loss).

Abbreviations: CALLY index, C-reactive protein-albumin-lymphocyte index; AC, anastomotic complications; PY, person-years; HR, hazard ratios; CI, confidence interval; SD, standard deviation; Ref, reference.

**Table S12. Subdistribution hazard ratios for the association between CALLY index and incident recurrence in competing risk-adjusted models in the Shanghai Sixth People's Hospital patient cohort (validation cohort)**

|  | **Recurrence events** | **Competing events** | **Unadjusted,**  **SHR (95% CI)** | **Adjusted for age and sex,**  **SHR (95% CI)** | **Main adjustment^a^,**  **SHR (95% CI)** | **Additional adjustment^b^,**  **SHR (95% CI)** |
| --- | --- | --- | --- | --- | --- | --- |
| Per 1 unit | 69 | 11 | 0.86 (0.81-0.92) | 0.85 (0.80-0.91) | 0.86 (0.80-0.92) | 0.86 (0.80-0.93) |
| Per 1 SD | 69 | 11 | 0.57 (0.43-0.74) | 0.54 (0.42-0.70) | 0.56 (0.43-0.73) | 0.56 (0.42-0.75) |
| CALLY index ≤ 2.55 | 40 | 4 | Ref | Ref | Ref | Ref |
| CALLY index > 2.55 | 29 | 7 | 0.40 (0.24-0.65) | 0.37 (0.22-0.61) | 0.35 (0.20-0.61) | 0.35 (0.20-0.60) |

Subdistribution hazard ratios (95% CI) for the association between CALLY index and recurrence. Analyses are performed in the Fine and Gray model.

^a^ Adjusted for age, sex, body mass index, hypertension, diabetes mellitus, cerebrovascular accident, tumor location, histological type, degree of differentiation, and TNM stage

^b^ Main adjustment + surgical information (duration of surgery, anastomosis site, intra-operative red blood cell transfusion, and intra-operative blood loss).

Abbreviations: CALLY index, C-reactive protein-albumin-lymphocyte index; SHR, subdistribution hazard ratios; CI, confidence interval; SD, standard deviation; Ref, reference.





**Figure S1. Flowchart of study enrollment**

Abbreviations: CALLY, C-reactive protein-albumin-lymphocyte; OS, overall survival; DFS, disease-free; AC, Anastomotic complications, SHSH, Shanghai Sixth People's Hospital; IINS, Immune-inflammatory-nutritional score; NLR, Neutrophil-to-lymphocyte ratio; PLR, Platelet-to-lymphocyte ratio.


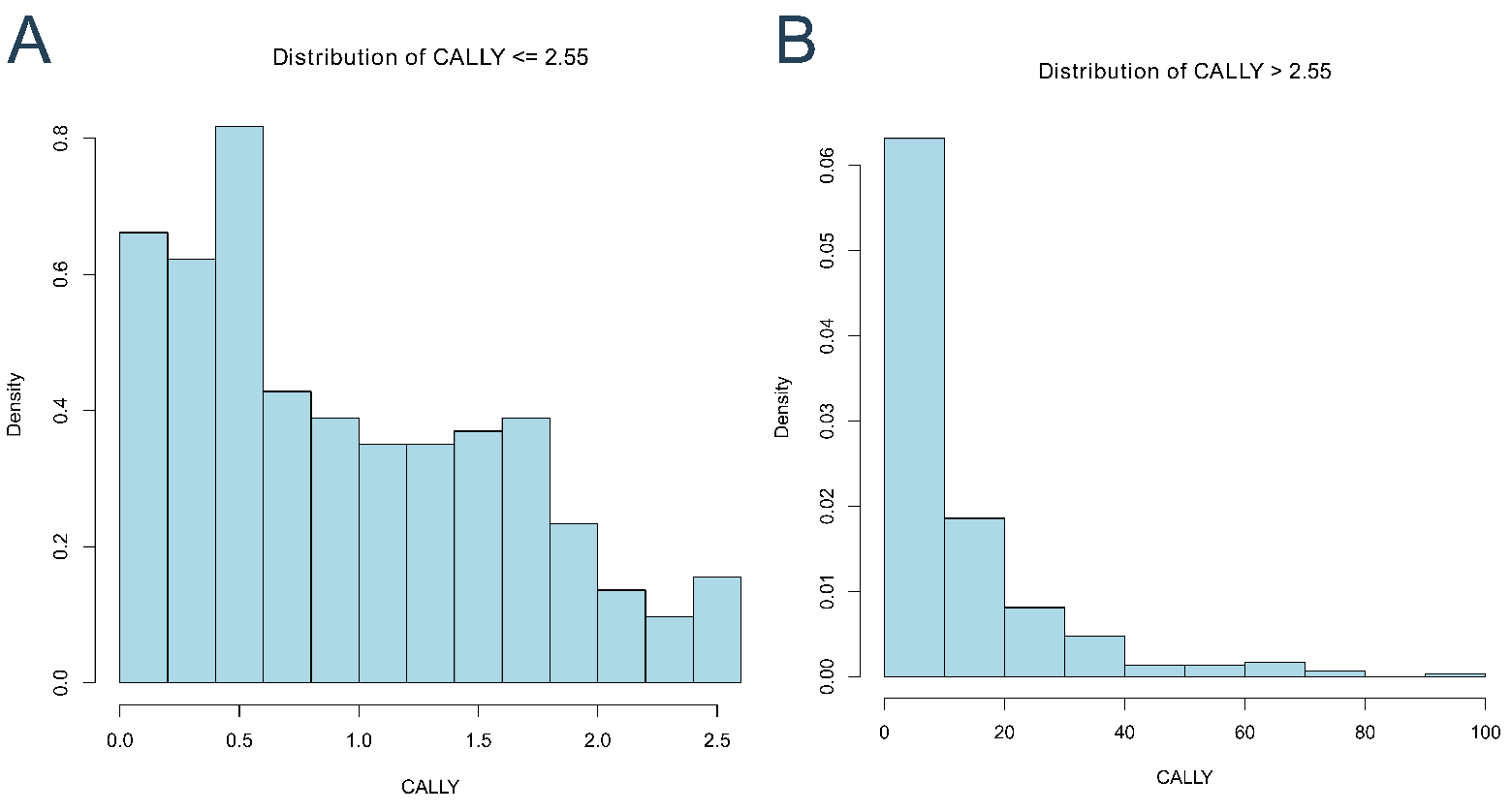


**Figure S2. Density distribution of CALLY index in the training study population**

Abbreviations: CALLY, C-reactive protein-albumin-lymphocyte.


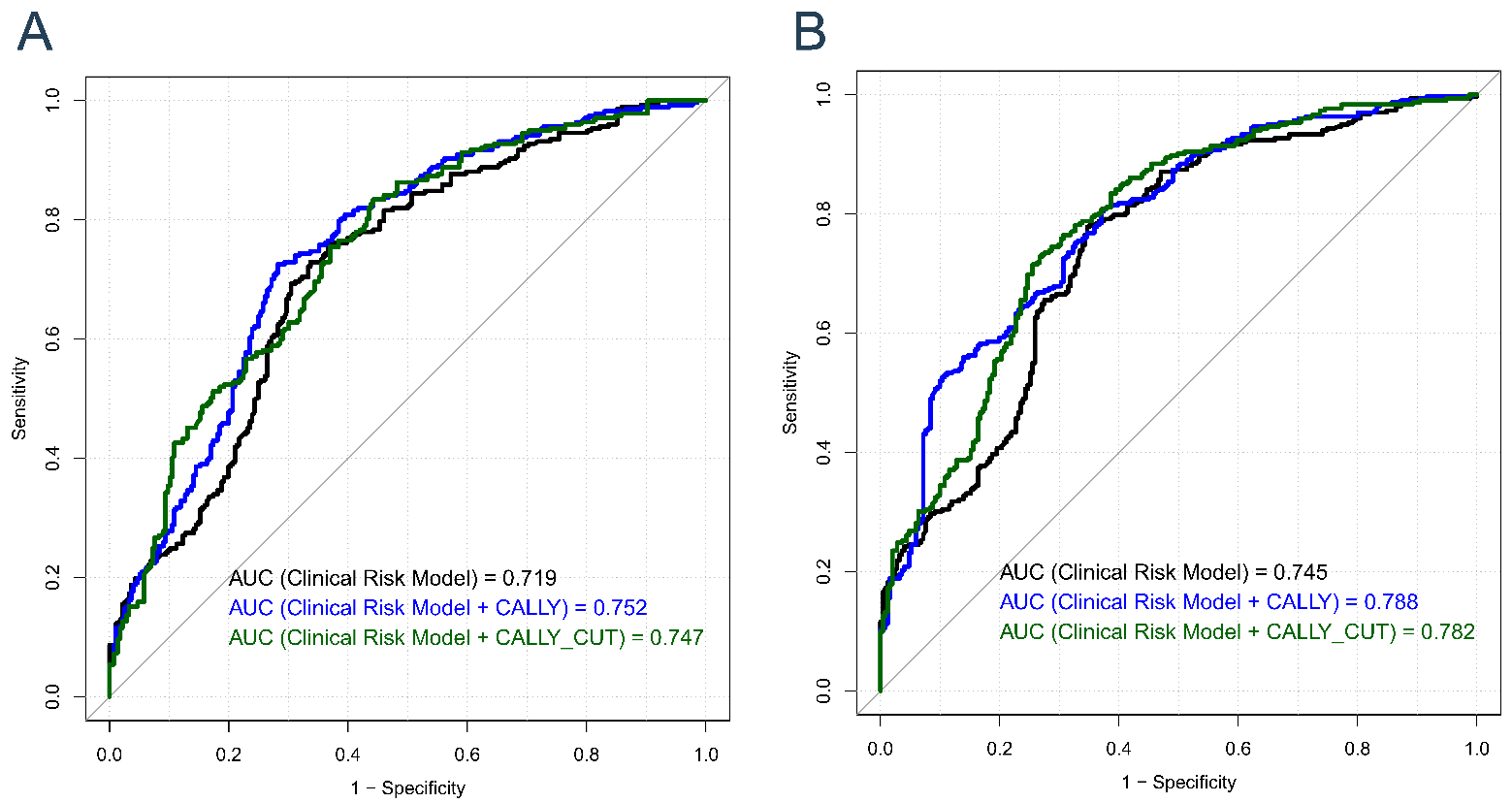


**Figure S3 The** **area under the receiver operating characteristic curve of clinical risk models combined or not combined with CALLY index in predicting OS and DFS**

Abbreviations: CALLY, C-reactive protein-albumin-lymphocyte; AUC, Area Under Curve; OS, Overall survival; DFS, Disease-free survival.


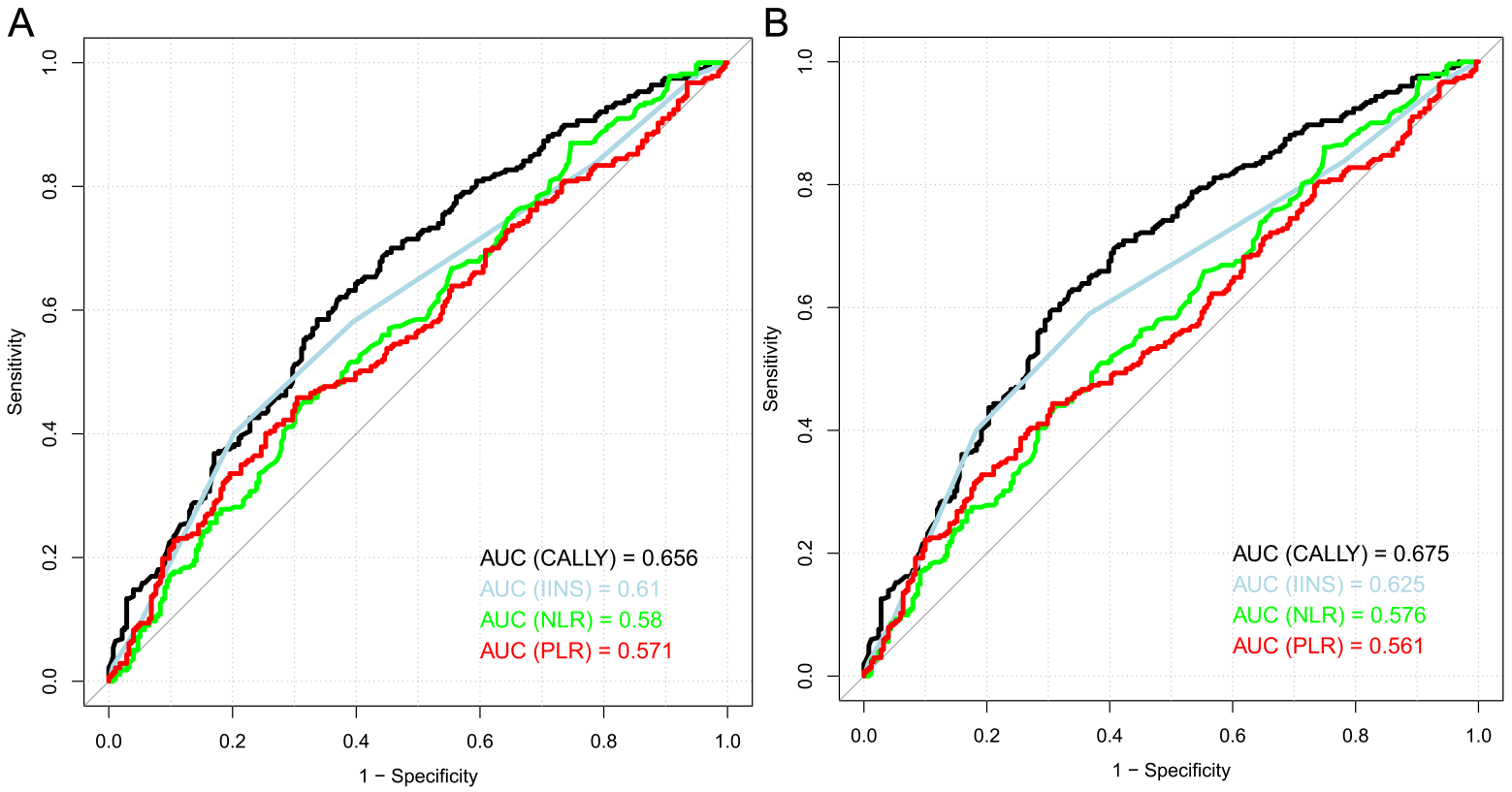


**Figure S4 Area under the receiver operating characteristic curve of biomarkers combined with clinical risk model**

Abbreviations: CALLY, C-reactive protein-albumin-lymphocyte; AUC, Area Under Curve; IINS, Immune-inflammatory-nutritional score; NLR, Neutrophil-to-lymphocyte ratio; PLR, Platelet-to-lymphocyte ratio.


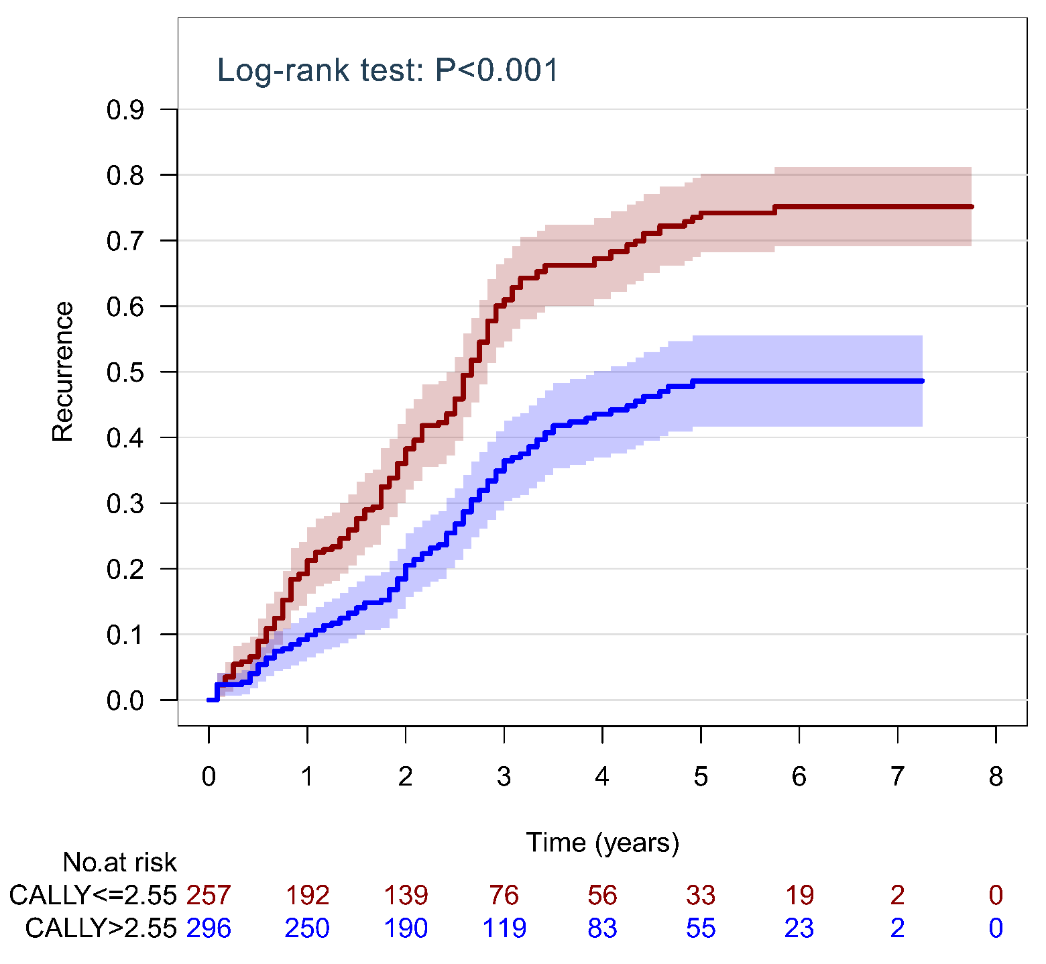


**Figure S5 Cumulative incidence curves for the rate of recurrence in patients with CALLY index ≤ 2.55 vs. CALLY index > 2.55 in the Shanghai General Hospital patient cohort (training cohort)**

Abbreviations: CALLY , C-reactive protein-albumin-lymphocyte.


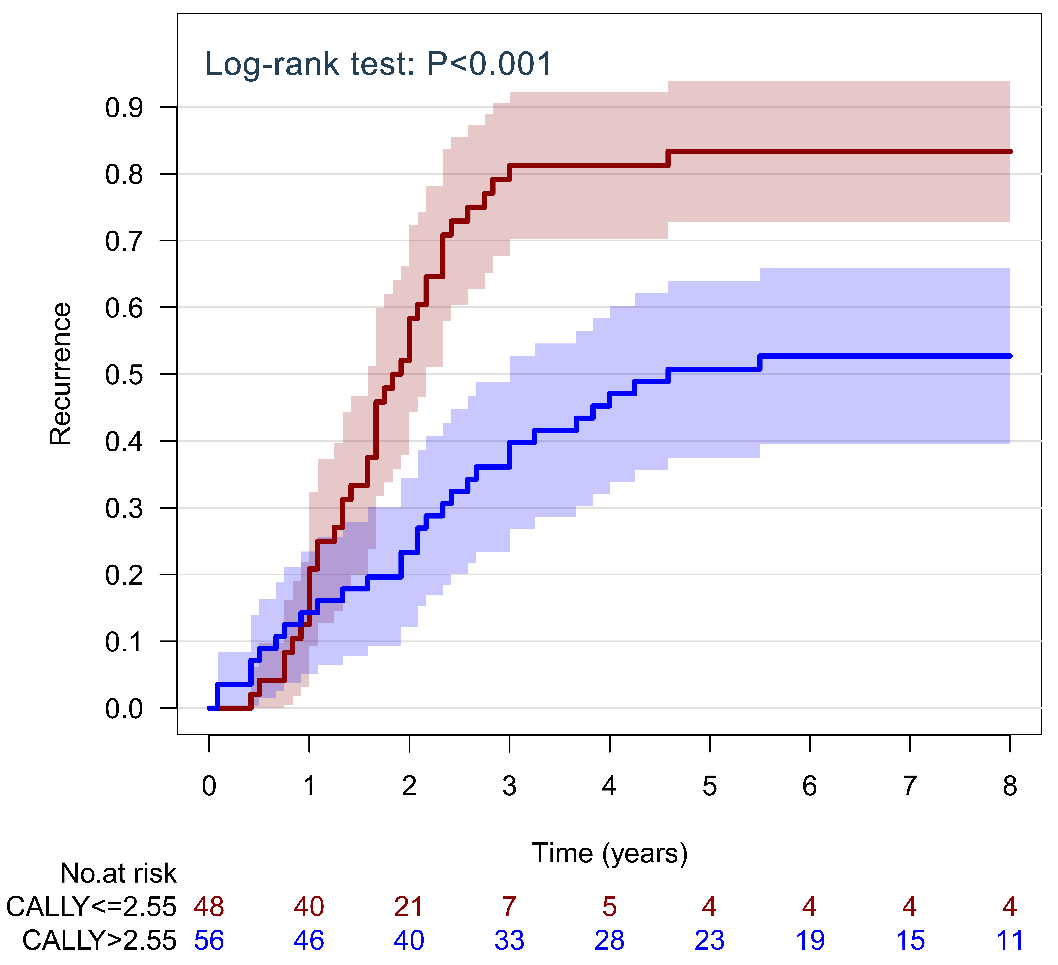


**Figure S6 Cumulative incidence curve for the rate of recurrence in patients with CALLY ≤ 2.55 vs. CALLY > 2.55 in the Shanghai Sixth People's Hospital patient cohort (validation cohort)**

Abbreviations: CALLY, C-reactive protein-albumin-lymphocyte.

**
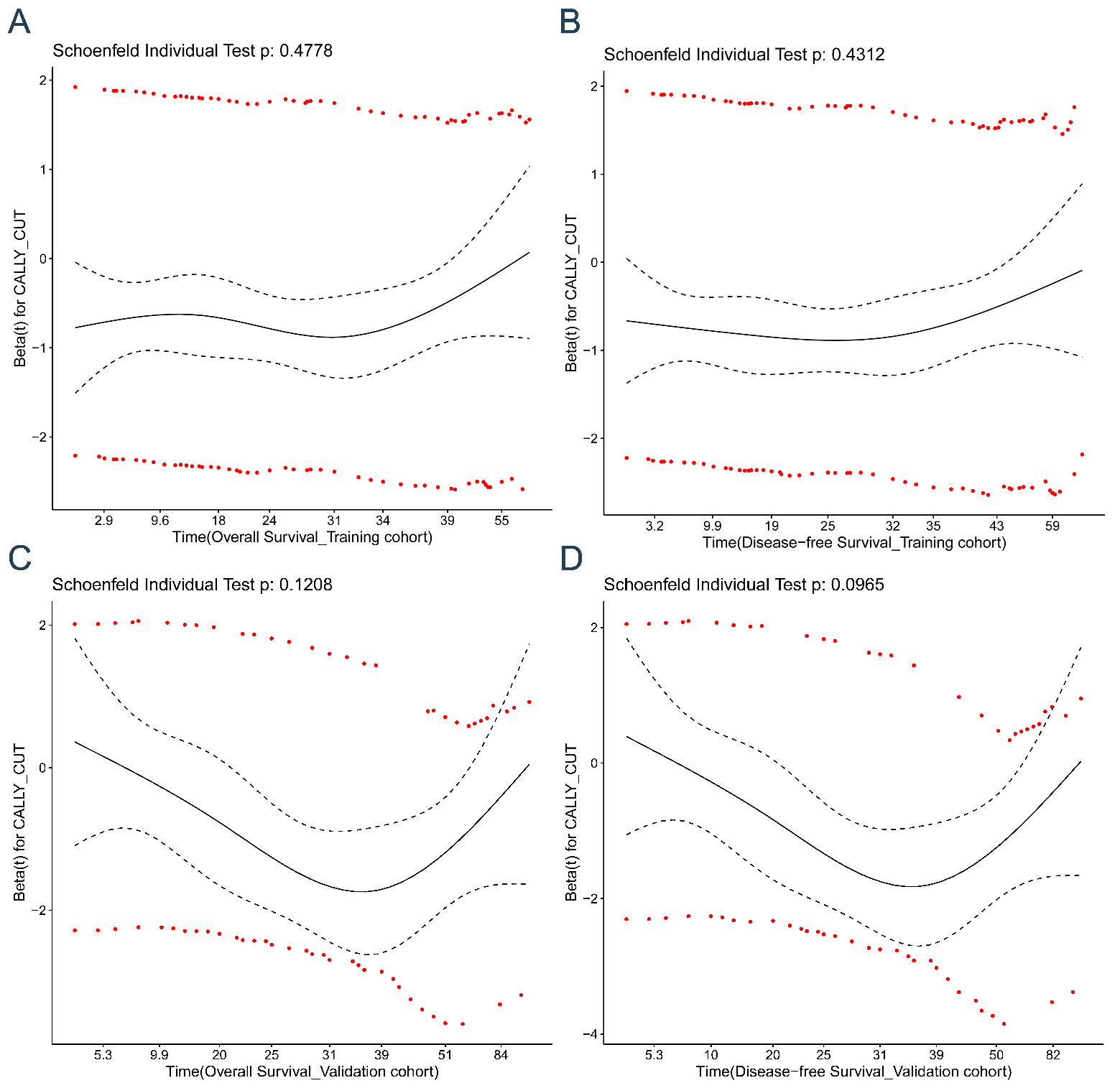
**

**Figure S7. Schoenfeld residuals plot of the CALLY index for OS/DFS over time**

Abbreviations: CALLY, C-reactive protein-albumin-lymphocyte.

**References:**

1. Ma J, Zhan C, Wang L, Jiang W, Zhang Y, Shi Y, et al. The sweet approach is still worthwhile in modern esophagectomy. ANN THORAC SURG. [Comparative Study; Journal Article; Research Support, Non-U.S. Gov't]. 2014 2014/5/1;97(5):1728-33.

2. Franke F, Moeller T, Mehdorn A, Beckmann JH, Becker T, Egberts J. Ivor-Lewis oesophagectomy: A standardized operative technique in 11 steps. INT J MED ROBOT COMP. [Journal Article]. 2021 2021/2/1;17(1):1-10.

3. Mu J, Gao S, Xue Q, Mao Y, Wang D, Zhao J, et al. Updated experiences with minimally invasive McKeown esophagectomy for esophageal cancer. WORLD J GASTROENTERO. [Journal Article; Research Support, Non-U.S. Gov't]. 2015 2015/12/7;21(45):12873-81.
